# Disruption of the *Lotus japonicus* transporter *LjNPF2.9* increases shoot biomass and nitrate content without affecting symbiotic performances

**DOI:** 10.1186/s12870-019-1978-5

**Published:** 2019-08-30

**Authors:** Stefano Sol, Vladimir Totev Valkov, Alessandra Rogato, Mélanie Noguero, Laura Gargiulo, Giacomo Mele, Benoit Lacombe, Maurizio Chiurazzi

**Affiliations:** 1 Institute of Biosciences and Bioresources, IBBR, CNR, Via P. Castellino 111, 80131 Naples, Italy; 2 BPMP, Univ. Montpellier, CNRS, INRA, SupAgro, Montpellier, France; 3 Istituto per i Sistemi Agricoli e Forestali del Mediterraneo, ISAFOM, CNR, Via Patacca 85, 80056 Ercolano, Italy

**Keywords:** Insertion mutants, Legumes, Nitrate distribution, Nitrate transport, Nitrogen use efficiency

## Abstract

**Background:**

After uptake from soil into the root tissue, distribution and allocation of nitrate throughout the whole plant body, is a critical step of nitrogen use efficiency (NUE) and for modulation of plant growth in response to various environmental conditions. In legume plants nitrate distribution is also important for the regulation of the nodulation process that allows to fix atmospheric N (N_2_) through the symbiotic interaction with rhizobia (symbiotic nitrogen fixation, SNF).

**Results:**

Here we report the functional characterization of the *Lotus japonicus* gene *LjNPF2.9,* which is expressed mainly in the root vascular structures, a key localization for the control of nitrate allocation throughout the plant body. LjNPF2.9 expression in *Xenopus laevis* oocytes induces ^15^NO_3_ accumulation indicating that it functions as a nitrate importer. The phenotypic characterization of three independent knock out mutants indicates an increased shoot biomass in the mutant backgrounds. This phenotype is associated to an increased/decreased nitrate content detected in the shoots/roots. Furthermore, our analysis indicates that the accumulation of nitrate in the shoot does not affect the nodulation and N-Fixation capacities of the knock out mutants.

**Conclusions:**

This study shows that LjNPF2.9 plays a crucial role in the downward transport of nitrate to roots, occurring likely through a xylem-to-phloem loading-mediated activity. The increase of the shoot biomass and nitrate accumulation might represent a relevant phenotype in the perspective of an improved NUE and this is further reinforced in legume plants by the reported lack of effects on the SNF efficiency.

**Electronic supplementary material:**

The online version of this article (10.1186/s12870-019-1978-5) contains supplementary material, which is available to authorized users.

## Background

Nitrate is one of the major forms of nitrogen (N) that plants acquire from the soil. Plants as sessile organisms, must cope with variable soil nitrate concentrations to optimize its uptake from surroundings, vacuolar storage/remobilization as well as distribution into different plant tissues. Besides, plants must perceive and elaborate internal nutritional status for deciphering N demand and consequently, decide how much nitrate to take up, store/assimilate and translocate for supporting an optimal plant growth. Plant transporters are crucial actors in this network of nitrate flux and the four protein families involved are: chloride channel (CLC), slowly activating anion channel (SLAC), nitrate/peptide transporter (NPF) and nitrate transporter (NRT2) with NPF and NRT2 playing a major role [1, 2].

Plants NPF families host a very high number of members with 53 known genes in *Arabidopsis thaliana* and 80 in *Oriza sativa*. NPF transporters generally exhibit a low affinity for nitrate, with the exception of AtNPF6.3 [3], MtNPF1.7 [4], MtNPF6.8 [5] and OsNPF6.5 [6], which display dual affinities. After uptake from the soil, the amount of nitrate assimilated in the root or transported to the shoot depends from different aspects such as spatial pattern of nitrate reductase (NR) in different plant tissues and organs [7], temperature [8], nitrate availability and light intensity [9, 10]. A number of NPF proteins have been involved in *A. thaliana* and *O. sativa* for balancing the upward and downward translocation of nitrate between roots and shoots. *AtNPF7.3* is a nitrate-induced gene that mediates xylem loading in root pericycle cells for nitrate translocation to the shoot [11]. The root-to-shoot nitrate transport is also mediated by *AtNPF2.3*, a constitutively expressed gene whose activity is observed only under conditions of salinity to prevent excessive nitrate allocation to the roots [12]. On the contrary the *AtNPF7.2* gene, expressed in xylem parenchyma cells, has been involved in retaining of nitrate in roots in adverse conditions [13]. The loading activity of AtNPF7.3 is also partially counteracted by AtNPF2.9 that mediates phloem loading and downward nitrate transport [14]. Furthermore, Hsu and Tsay [15] have reported that the phloem localized AtNPF1.1 and AtNPF1.2 are involved in nitrate redistribution in the leaves. More recently, the biochemical characterization of the tonoplast-located AtNPF5.11, AtNPF5.12 and AtNPF5.16 indicates a nitrate efflux activity in *Xenopus laevis* that could be involved in the control of nitrate partitioning between roots and shoots [16]. In *O. sativa* the *OsNPF2.4* gene expressed in epidermis, xylem parenchyma and phloem companion cells has been involved both in nitrate acquisition from external sources into roots and upward transport from roots to shoots [17], two functions that have been also associated to the OsNPF6.5 transporter [6]. On the other side, the function of xylem nitrate unloading and control of root-to-shoot nitrate transport seems to be shared in *O. sativa* by OsNPF2.2 that is also involved in the development of vasculature [18].

It is well known that leguminous plants have evolved the ability to establish a symbiotic interaction with the soil bacteria rhizobia (SNF), leading to root infection, nodule organogenesis, colonization of the new organ and fixation of atmospheric nitrogen. The occurring of this symbiotic process adds a further reason of interest to the functional characterization of the nitrate transporters in legumes, as nitrate is known to regulate all the different steps of nodulation by acting both as a nutrient and a signal [19–23]. In particular, the nodule formation capacity is locally inhibited in legume plants exposed to increasing concentrations of external nitrate [22] as well as systemically inhibited in N-fed plants [23, 24]. This implies that the systemic control of SNF due to the N-nutritional status of legumes may have a cross-talk with the pathways governing NUE. This aspect must be taken in consideration as the strategies aiming to improve NUE in legumes could be conflictual for an efficient symbiotic interaction.

Besides, a role on the control of the SNF might be encompassed by the capacity of NPF proteins to transport different substrates than nitrate such as hormones, dicarboxylic acids, dipeptide, amino-acids [25–31]. Nevertheless, the study of nitrate transporter in legumes has received little attention so far and few reports have been published on analyses of NPF families in legumes [5, 32–35]. The NPF involvement on the nodulation process has been reported only for *MtNPF1.7* controlling the nodule meristem formation and invasion [36, 37] and *LjNPF8.6* that plays a role in the regulation of nodule functioning [35].

Here we report the functional characterization of the *LjNPF2.9* gene encoding for a protein that show nitrate transport capacity in *Xenopus laevis* and it is involved in the control of nitrate distribution in Lotus plants playing a role in the downward transport toward root tissue. The higher nitrate content detected in the shoots of three independent knock-out mutants does not interfere with their nodulation and N-fixing capacities.

## Results

### NPF identification in *L. japonicus*

A provisional list of 39 NPF members from *L. japonicus* [38]*,* which was not included among the 31 plant species described in Leran et al., [1] has been already reported. A reiterated blast search against the enhanced *L. japonicus* genomic assembly (release 3.0), has allowed now the identification of a larger, *LjNPF* family composed of 86 members (Additional file 1: Table S1 and Additional file 2: Table S2). The phylogenetic tree obtained with the maximum parsimony method and based on the alignment of the *A. thaliana* and *L. japonicus* amino acid NPF sequences indicates the expected distribution among the 8 clades identified by Leran et al., [1] (Additional file 3: Figure S1). As reported by Leran et al., [1] the nomenclature has been assigned by a two letters code, where the first identifies the clade number and the second differentiates the genes within the *L. japonicus* family and does not reflect orthologous relationships (Additional file 1: Table S1). Interestingly, the size of the whole LjNPF family and of the 8 identified clades close the numbers of NPFs found in the other diploid model legume *Medicago truncatula* [1].

### *LjNPF2.9* is mainly expressed in roots vascular tissue

Lj2g3v1349210.1 (genomic assembly build 3.0; http://www.kazusa.or.jp/lotus/) has been sub-classified in the clade 2 and re-named *LjNPF2.9* (Additional file 1: Table S1), encoding for a 635 amino acid protein with a predicted molecular mass of 70.4 kDa. LjNPF2.9 as most of the NPF proteins, is predicted to contain 12 transmembrane domains with a 95 amino acid long cytoplasmic loop between domains 6 and 7 (Additional file 4: Figure S2).

We have previously reported the preliminary molecular characterization of *LjNPF2.9* (Chr2CM0608.1290.r2.m in the previous genomic assembly release 2.5) by analysing its transcriptional profile in root tissue after nitrate provision at different concentrations [31]. A time-course experiment did not reveal significant transcriptional responses in root tissues neither after shift to both low and high nitrate external concentrations, nor at early times after *Mesorhizobium loti* inoculation [32].

**Fig. 1:**
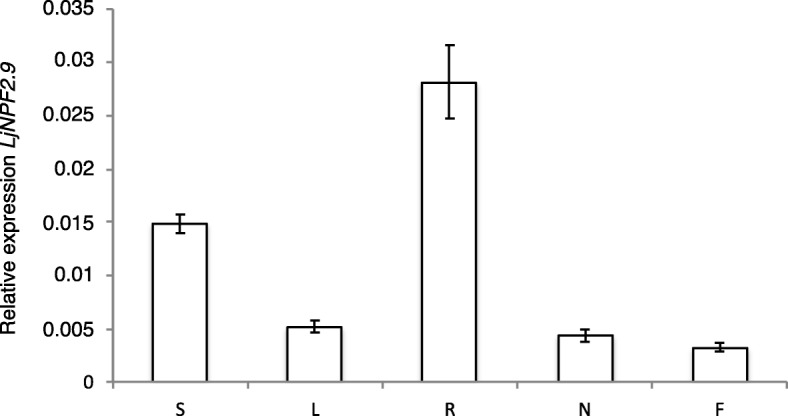
*LjNPF2.9* expression in different organs. RNAs are extracted by wild type plants grown on B5-Gamborg derivative medium with 1 mM KNO_3_ as N source, at 3 weeks after *M. loti* inoculation. Mature flowers have been obtained from Lotus plants grown in the growth chamber. S=Stems; L = Leaves; R = Roots; N=Nodules; F=Flowers. Data bars represent the mean and standard deviations of data obtained with RNAs extracted from three different sets of plants and 3 RT-qPCR experiments

The profile of expression of *LjNPF2.9* has been further characterized by analyzing the distribution of the transcript in different organs of *L. japonicus* by qRT-PCR. Seedlings germinated on Gamborg-B5 derivative medium with 1 mM KNO_3_ as N source, have been inoculated with *M. loti* and RNAs extracted after 3 weeks from different organs. The *LjNPF2.9* is ubiquitary expressed in *L. japonicus* organs with the highest level of expression detected in roots and stems (Fig. 1).

**Fig. 2:**
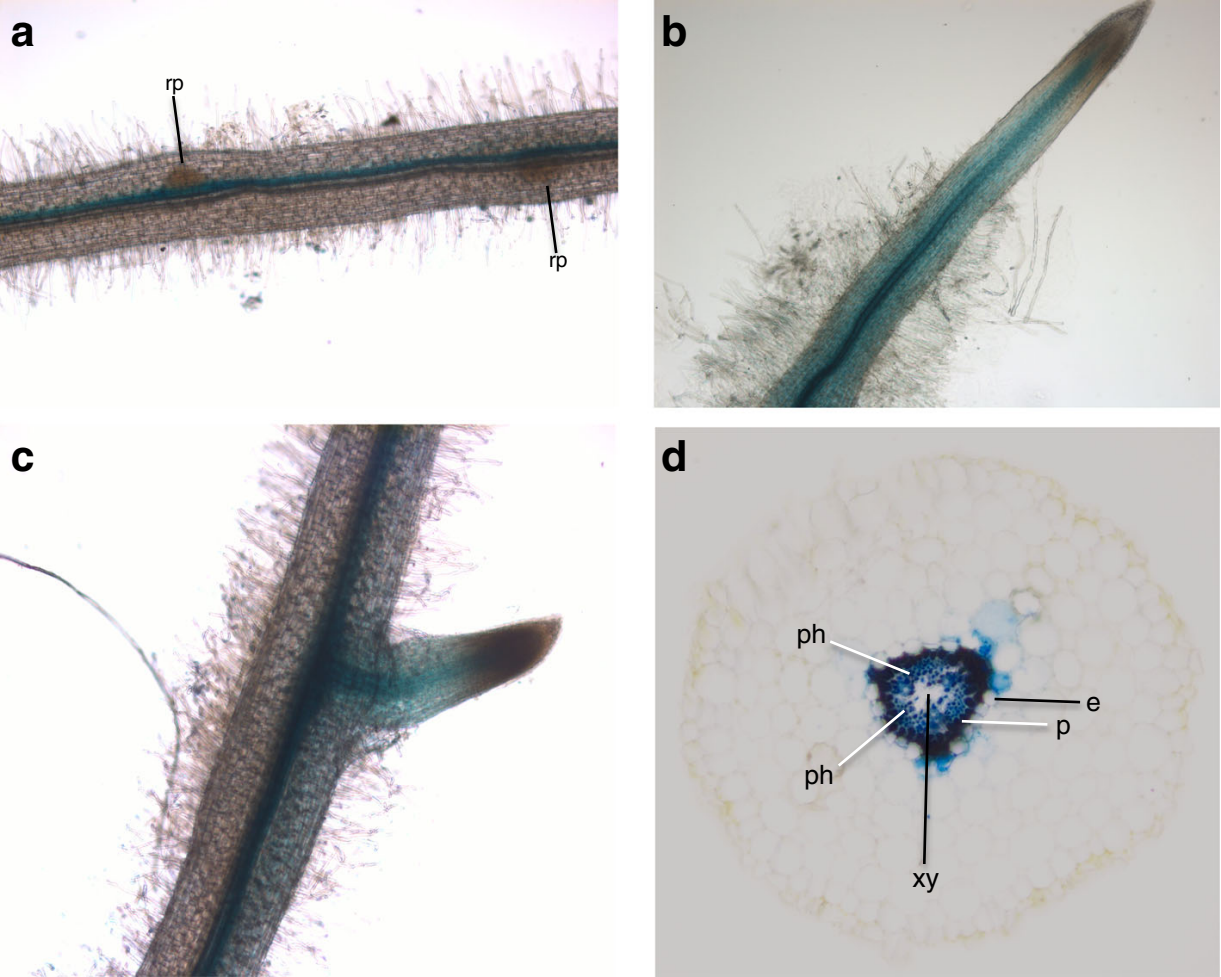
Representative spatial profile of the *LjNPF2.9* promoter activity in transgenic hairy roots. **a**-**c** GUS activity in vascular bundle of mature (**a**) and young root regions (**b** and **c**). **d** cross section of a stained hairy root. Rp = root primordium; e = endodermis; p = pericycle; ph = phloem; xy = xylem

To gain further information about the profile of *LjNPF2.9* expression in root tissues, a PCR fragment extending up to 1038 bp upstream of the ATG and including the first ten LjNPF2.9 codons has been amplified to obtain a translational fusion with the *gus*A reporter gene. *Lotus* composite plants obtained upon transformation with *Agrobacterium rhizogenes* have been obtained and GUS activity tested in hairy roots. GUS staining is confined to vascular structures of primary and lateral roots and no activity could be observed in meristematic regions of primary and secondary roots as well as in root primordia (Fig. 2a-c). The root cross section in Fig. 2d confirms a GUS activity limited to the root vascular bundle, with a more intense staining in the pericycle cell layer and phloematic regions.

### Isolation of LORE1-insertion null mutants and phenotypic analyses of biomass parameters

**Fig. 3:**
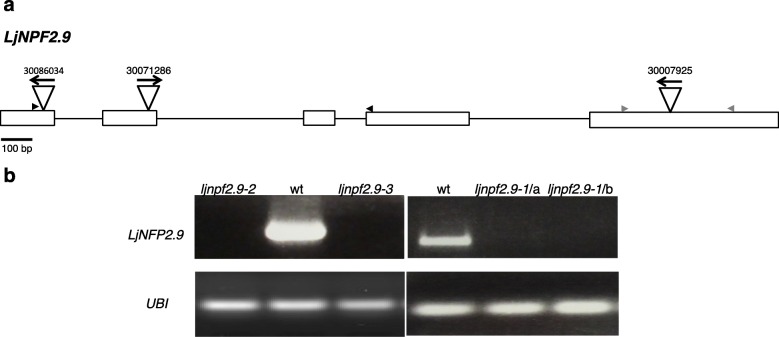
**a** exon/intron organization of the *LjNPF2.9* gene. Insertion sites, couple of primers (black and grey; Additional file 8: Table S4) used for expression analyses and relative orientations of the LORE1 retrotransposon element in the 30,086,034, 30,071,286 and 30,007,925 lines are indicated; **b** expression analysis of the *LjNPF2.9* gene. Total RNAs isolated from root tissues of the wild type and *ljnpf2.9* mutants obtained from the three different LORE1 lines has been used for RT-PCR analysis (two different *ljnpf2.9–1* mutants, a and b are shown)

**Fig. 4:**
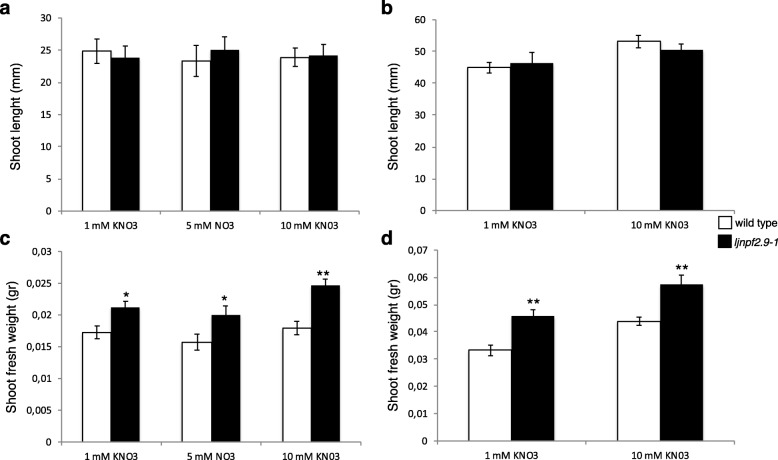
Shoot biomass parameters of wild type and *ljnpf2.9–1* mutants grown on different KNO_3_ conditions. **a** and **b** shoots length at 2 and 3 weeks after sowing; **c** and **d** fresh shoots weight at 2 and 3 weeks after sowing. Plant genotypes and KNO_3_ concentrations are indicated. Data bars represent the mean and standard errors obtained from 3 independent experiments (10 plants per experiment). Asterisks indicate significant differences with wild type values: *p < 0.02; ** < 0.0007

**Fig. 5:**
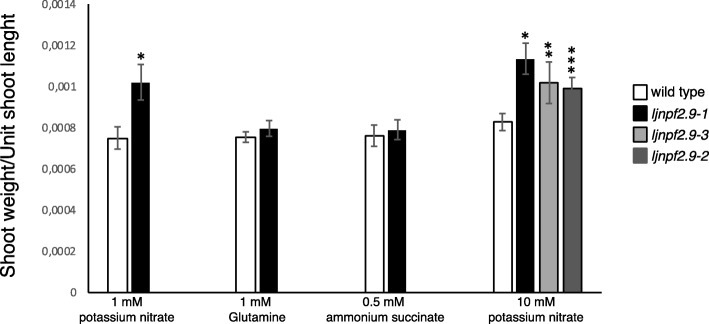
Shoots weight/unit length of wild type and *ljnpf2.9* mutants grown in the presence of different N sources. Plant genotypes and N sources concentrations are indicated. 0.5 mM ammonium succinate (NH_4_OOC(CH_2_)COONH_4_) is equivalent to 1 mM ammonium. Data bars represent the mean and standard errors obtained from 3 independent experiments (10 plants per experiment). Asterisks indicate significant difference with wild type values: *p < 0.0001; **p < 0.001; ***p < 0.002

To determine the in vivo function of *LjNPF2.9*, three independent LORE1 insertion mutants have been isolated from the *L. japonicus* LORE1 lines collection [39–41]. Lines 30,086,034, 30,071,286 and 30,007,925, bearing retrotransposon insertions in the first, second and fifth exon (Fig. 3a), have been genotyped by PCR. Shoot cuts of homozygous plants for the insertion event into the *LjNPF2.9* gene have been cultured in axenic conditions and then transferred to the growth chamber for seeds production. Endpoint RT-PCR analyses conducted with primers bracketing the insertion site of homozygous plants from lines 30,086,034, 30,071,286 and 30,007,925 have revealed no detectable *LjNPF2.9* full size mRNA in roots and hence, considered null mutants (Fig. 3b). The homozygous plants of the line 30,007,925 (from here-on called *ljnpf2.9–1*) have been obtained first and initially characterized for biomass phenotypes in different growth conditions. Two individual homozygous mutant plants for this line have been selected for analyses (*ljnpf2.9–1*/a and *ljnpf2.9–1*/b in Fig. 3b) and because their growth phenotypes did not significantly differ, the data obtained with the selected individual mutants have been pooled in this study. *Ljnpf2.9–1* mutants grown in the presence of 1 mM, 5 mM and 10 mM KNO_3_ concentrations have been scored for shoot length and fresh weight at two and 3 weeks after sowing. The shoot length of *ljnpf2.9–1* mutants did not show any significant difference when compared to wild type plants (Fig. 4a, b), whereas a striking difference was detected in the fresh shoots weight (Fig. 4c, d). The shoot fresh weight of *ljnpf2.9–1* mutants are significantly increased (20–28%) compared to wild type and the observed increase is maintained in the whole range of external KNO_3_ concentrations (Fig. 4c, d). Therefore, to confirm that the *LjNPF2.9* mutation is really responsible of the increased shoot weight, the same phenotype has been tested for the mutants obtained from lines 30,071,286 and 30,086,034 (here-on called *ljnpf2.9–2* and *ljnpf2.9–3*) grown in the presence of 10 mM KNO_3_. As shown in Fig. 5, a significant increase of the shoot weight/length ratio has been scored in all the tested *ljnpf2.9* lines, hence indicating that the LORE1 insertions in the *LjNPF2.9* gene are the causal mutations of the observed phenotype. The causal relationship between knock out mutations in the *LjNPF2.9* gene and the observed phenotypes are confirmed by the analyses conducted on heterozygous segregants for the LORE1 insertion in the *LjNPF2.9* gene, obtained from two out of the three insertion lines analyzed in this work. These heterozygous plants don’t display the higher shoot weight/length ratio and are not distinguishable from wild type plants (data not shown). Importantly, the increased shoot weight/length ratio of the *ljnpf2.9* mutants has been observed only when KNO_3_ is used as N source, as the same phenotype is not detected neither on glutamine nor ammonium succinate media (Fig. 5).

**Fig. 6:**
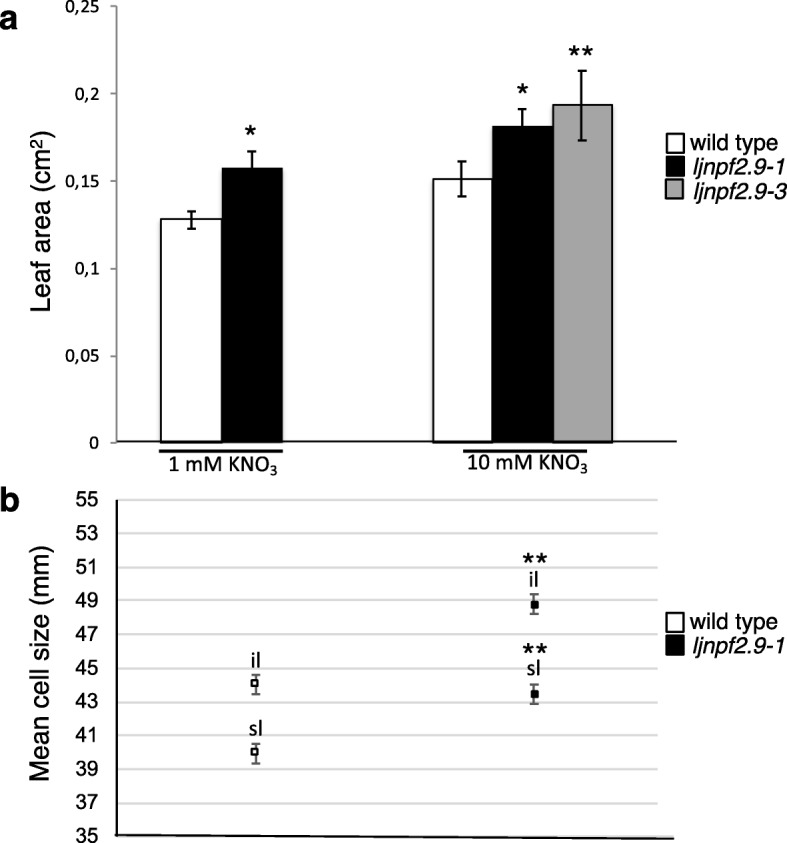
Morphometric measurements of leaves of wild type, *ljnpf2.9–1* and *ljnpf2.9–3* mutants grown in the presence of 1 mM and 10 mM KNO_3_. **a** leaf area (*n* = 60). Correspondent trifolia from 20 plants for each genotype have been analyzed. Bars represent mean and standard errors; **b** cell size (equivalent diameter) of leaves (*n* = 18). Correspondent trifolia from 3 plants for each genotype have been detached and analyzed. sl = superior lamina; il = inferior lamina. Bars represent the common standard error from two factors ANOVA. Different plant genotypes are indicated. Asterisks indicate significant differences (*p < 0.001;** *p* < 0.0001)

In order to define the shoot anatomical trait associated to the increased weight scored in the presence of KNO_3_ (Figs. 4 and 5), we have also compared morphometric data of wild-type and *ljnpf2.9* mutants. Wild type and mutant plants have been grown for 2 weeks after sowing, in the presence of 10 mM nitrate as sole N source and fully expanded, detached leaves from 1st 2nd and 3rd trifolia, were collected for the analysis. The average leaves area in the *ljnpf2.9–1* and *− 3* plants is significantly increased by 17 to 22% compared to wild type (Fig. 6a and Additional file 5: Figure S3). Furthermore, to test whether the increased leaf size in the mutant lines is a result of abnormal cell expansion or increased cell number, we have examined the cell size of both adaxial and abaxial lamina of epidermis cell layer in correspondent trifolia of wild type and *ljnpf2.9–1* plants. The measures reported in Fig. 6b indicate that epidermal cells of leaves of *ljnfp2.9–1* mutants are larger than those in wild type suggesting an effect on cell size control (Fig. 6b). In the Additional file 6: Figure S4 is shown an example of the leaf morphometric analysis performed on wild type and *ljnfp2.9–1* mutants correspondent trifolia. The comparison of the epidermis cells covering the same wild type and *ljnfp2.9–1* leaf areas, allows to visualize the clear-cut increased size observed in the leaf mutants. The measures and statistical analyses are reported in the Additional file 7: Table S3. The increased cell size scored in the leaf mutants (Fig. 6b) is confirmed by the cells counting indicating a lower number of cells in the epidermis of leaf mutants per square area analyzed.

### LjNPF2.9 is a nitrate transporter

**Fig. 7:**
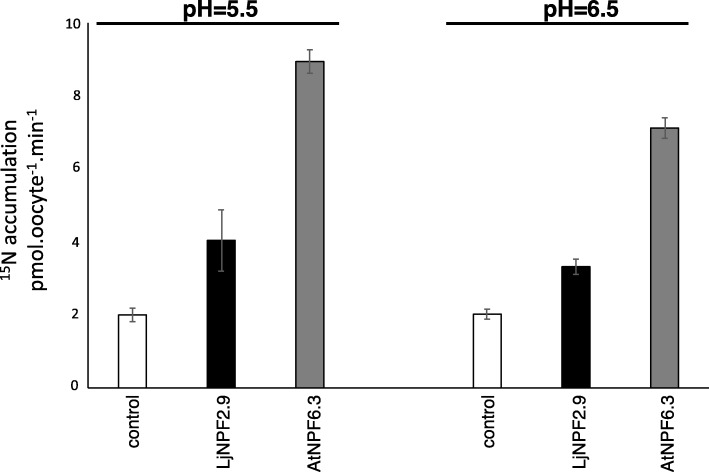
Functional expression of *LjNPF2.9* in *Xenopus laevis* oocytes in 10 mM external nitrate at pH 5.5 and 6.5. Nitrate accumulation in control oocytes injected with water (white bars), with complementary RNAs expressing LjNPF2.9 (black bars) and AtNPF6.3 (grey bars) (*n* = 5–8). Values are means ± SE

A low affinity transport capacity has been reported for 6 out of the 14 members of the *A. thaliana* NPF2 clade [1, 42]. In order to check whether *LjNPF2.9* encodes a nitrate transporter, in vitro-transcribed *LjNPF2.9* complementary RNA (cRNA) has been injected into *Xenopus laevis* oocytes for functional assay. Two days after cRNA injection, the oocytes have been tested for nitrate ^15^NO_3_ uptake activity at pH 5.5 and 6.5 at a concentration of 10 mM. *LjNPF2.9* cRNA-injected *Xenopus laevis* oocytes have been compared to the *AtNPF6.3* injected ones. Both batches of oocytes display NPF-dependent ^15^NO_3_ accumulation at 10 mM, which is only slightly reduced at pH 6.5 (Fig. 7). The uptake activity displayed by LjNPF2.9 in the *Xenopus laevis* heterologous system suggests a physiological low affinity NO_3_ transport activity carried out by this transporter in Lotus roots.

### *ljnpf2.9* mutants have an increased nitrate content in the shoots

**Fig. 8:**
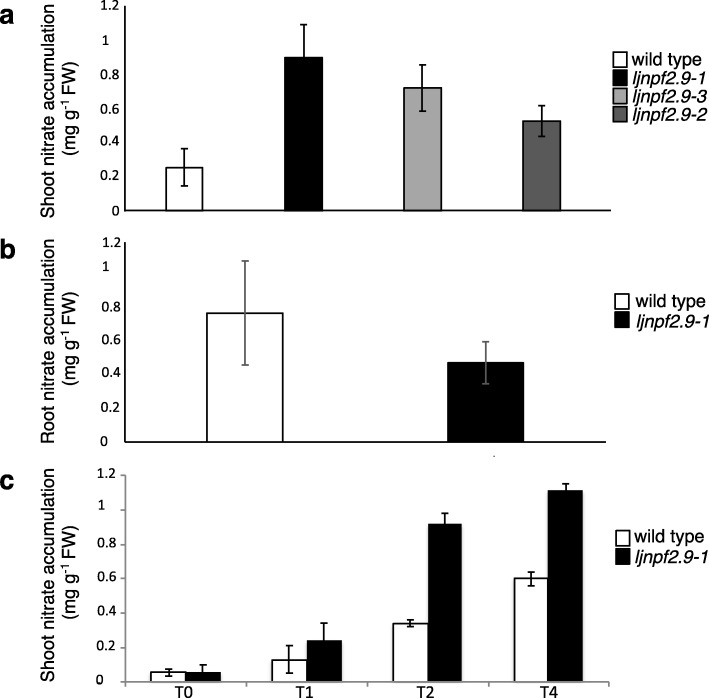
**a** and **b** nitrate content of shoots and roots of wild type and *ljnpf2.9* mutants. Plants are grown on 10 mM KNO_3_ and analyzed 3 weeks after sowing; **c** time course of the nitrate content in shoots of wild type and *ljnpf2.9–1* mutants. Plants grown for 2 weeks on glutamine 1 mM are transferred on 10 mM KNO_3_ and analyzed at different time points (0, 1, 2 and 4 days). Data bars represent means and SE from three independent samples (10 plants per sample). Bars corresponding to wild type and mutants plants are indicated

The nitrate transport capacity observed in *Xenopus laevis* oocytes as well as its localized expression in the root pericycle and phloem, prompted us to speculate that *LjNPF2.9* could be involved in the nitrate distribution between roots and shoot, a function that has been associated to an increased shoot biomass in *A. thaliana* [14]. We have compared the shoot nitrate content in wild type and *ljnpf2.9* mutant plants transferred 4 days after sowing on B5 derived medium with 10 mM KNO_3_ as the sole N source. The nitrate content has been scored 3 weeks after transfer. The leaves of the *ljnpf2.9* mutants show a 2–3.5 fold increased nitrate content when compared to wild type plants (Fig. 8a) and this phenotype is strikingly reverted in root tissues (Fig. 8b). This increased nitrate content in mutant leaves has been confirmed in a time course experiment where syncronyzed *L. japonicus* seedlings grown in the presence of glutamine 1 mM for 2 weeks are shifted on 10 mM KNO_3_ for 1, 2 and 4 days. The results shown in Fig. 8c indicates a progressive accumulation of nitrate in the leaves of both wild type and *ljnpf2.9–1* plants with a significant increased nitrate content in the shoots of the mutated genotypes.

### The nitrate-dependent nodulation inhibitory pathway is not altered in the *ljnpf2.9* mutants

**Fig. 9:**
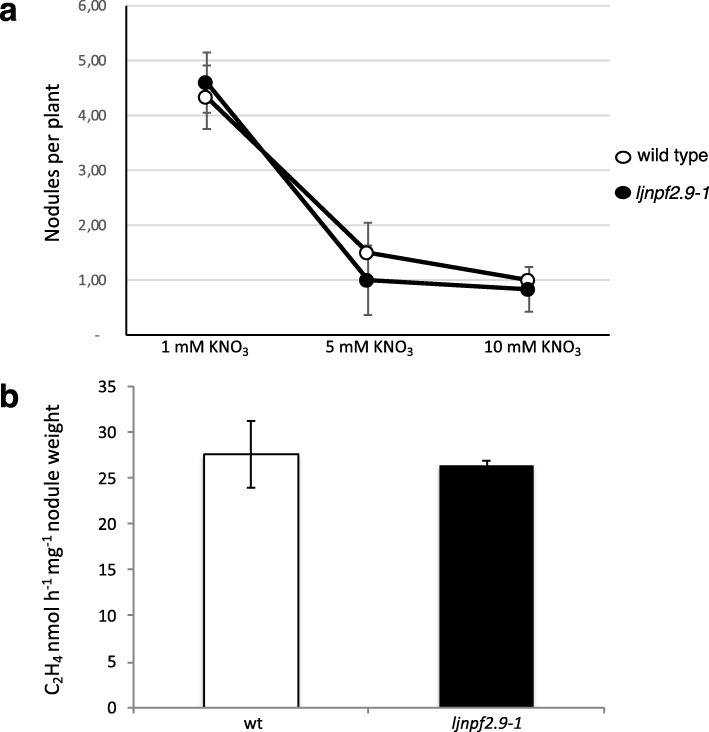
Symbiotic phenotypes of wild type and *ljnpf2.9–1* mutants. **a** number of nodules per plant. Mature nodules have been scored at 4 weeks post inoculation with *M. loti*. KNO_3_ concentrations are indicated; **b** acetylene reduction activity (ARA) per fresh nodule weight. Plants have been grown in the presence of 1 mM KNO_3_ and nodules analyzed at 4 weeks post infection. Plant genotypes are indicated. Data bars represent the mean and standard errors obtained from 3 independent experiments (10 plants per experiment)

High external nitrate concentrations exert an inhibitory action on nodulation by acting both as signal (local effect) and nutrient (systemic effect) [19, 21, 22, 24, 43, 44]. In the same way, high external nitrate concentrations are perceived quickly by nodulated legume plants, exerting an inhibitory effect on nitrogenase activity [45]. In order to check whether the nitrate accumulation detected in the shoots of *ljnpf2.9* mutants could affect the concentration-dependent inhibitory effects of external nitrate on nodule formation and functioning, we have compared nodule numbers and nitrogenase activity of wild type and mutant plants grown in a range of different external nitrate concentration. The results shown in Fig. 9a indicate an identical curve of nodule formation inhibition in the presence of progressively increased external nitrate concentrations. In the same way, the acetylene reduction activity (ARA) assay shown in Fig. 9b did not reveal significant difference on the N-fixation activity between wild type and the different mutated genotypes.

## Discussion

Nitrogen use efficiency (NUE; kg yield/applied kg N) is a crucial agronomic trait that must be improved in crop cultivations as in average less than 50% of the large amount of N fertilizers provided to agricultural lands to reach maximum yield are used by plants [46, 47]. About half of the applied N is lost to the air and water, or bound to the soil with dramatic anthropogenic consequences on pollution of aquatic environments, groundwaters and atmosphere. NUE is composed of two main components: i) uptake efficiency, the ability of plants to take up a given mineral nutrient from the soil; ii) utilization efficiency, the ability of plants to use the mineral nutrient such as nitrate to produce biomass. However, many specific physiological traits can play important roles in this complex network such as the distribution and allocation of nitrate throughout the whole plant body. In particular, a higher NUE has been associated to the promoted allocation of nitrate to aerial parts of plants [48] and recently many reports have highlighted the potential role of NPFs for the improvement of the NUE in crops [49–51].

We report here the functional characterization of a member of the *L. japonicus* NPF family, LjNPF2.9, which plays a positive role in the downward transport of nitrate in roots as well as the effects of knock out mutations on the symbiotic nitrate-dependent pathways controlling nodule formation and functioning.

The phenotypic analyses have been conducted on three independent *ljnpf2.9* mutants (Fig. 3) isolated from the collection of *L. japonicus* LORE1 lines [39–41], which display identical phenotypes. A striking increase of the shoot weight has been recorded in mutant plants grown in the presence of concentrations of potassium nitrate ranging from 1 mM to 10 mM (Fig. 4) and this improved shoot biomass phenotype is not observed in the presence neither of glutamine nor ammonium succinate as sole N sources (Fig. 5). Furthermore, *ljnpf2.9* mutants display an inverse correlation of nitrate content in shoot and roots when compared to wild type plants with an increased and reduced amount of accumulated nitrate in shoot and roots, respectively (Fig. 8), indicating that LjNPF2.9 plays a role for the balancing of root-to-shoot nitrate allocation by mediating the downward transport to roots. It is noteworthy that the increased shoot biomass of the *ljnpf2.9* mutants is a very intriguing phenotypic trait as allocation of more NO_3_^−^ to shoots has been associated with higher NUE in plants [48]. The physiological meaning of the nitrate-dependent phenotypes displayed by the *ljnpf2.9* mutants is further reinforced by the biochemical characterization carried out in *Xenopus laevis* oocytes, where the *LjNPF2.9* expression triggers a nitrate transport capacity in the presence of high nitrate concentration (Fig. 7). This transport capacity is consistent with the presence of the ExxER/K motif, located in the first transmembrane helix of LjNPF2.9 (Additional file 3: Figure S1), which differs members of clade 2 operating as symporters (NPF2.8–2.14 in *A. thaliana*) from the ones belonging to the subclade NPF2a (NPF2.1–2.7 in *A. thaliana*), displaying a passive transport capacity in heterologous systems [52, 53]. Although we cannot exclude that LjNPF2.9 might also transport other substrates than nitrate, the nitrate dependent growth phenotype (Fig. 5) and the altered nitrate content detected in the shoot and root tissues of *ljnpf2.9* mutants (Fig. 8) can be clearly associated to the uptake activity observed in the *Xenopus laevis* oocytes injected with the *LjNPF2.9* cRNA (Fig. 7). Our phenotypic characterization also includes the analyses of plant anatomical leaf traits as it is known that these may change in response to N availability [54, 55]. The analyses reported in Fig. 6 indicates that the increase of shoot biomass observed in the presence of nitrate is associable to cell size enlargement. The molecular mechanisms that may link the shoot nitrate content increase and cell expansion in *L. japonicus* have not been investigated in this work but nitrate-induced pathways such as carbon-metabolism and/or cytokinin responsive genes might be involved [56–58].

The increased nitrate accumulation observed in the shoots of the *ljnpf2.9* mutants and the reversed reduced nitrate content in root tissues could be either explained by a xylem unloading or phloem loading functions of LjNPF2.9. In *A. thaliana* the *AtNPF7.2* gene (AtNRT1.8) is induced in roots by nitrate and expressed in xylem parenchyma cells where it controls nitrate removal from xylem sap [13, 59]. Disruption of AtNPF7.2 alters nitrate distribution in plant tissues in the presence of cadmium (decreased root/shoot nitrate content ratio) suggesting a role in the retaining of nitrate in roots in stress conditions [13]. A similar reduced root/shoot nitrate content has been also reported for the *atnpf2.9* mutants [14]. *AtNPF2.9* (*AtNRT1.9*) is slightly induced by nitrate and expressed in the companion cells of root phloem where it acts as a facilitator of nitrate loading as indicated by the decreased nitrate content detected in the mutant root phloem exudates. AtNRT2.9 is hence responsible of the downward transport by loading nitrate into root phloem, where apoplastic nitrate source for such loading activity may come from efflux of vascular parenchyma cells or leakage of xylem stream. Strikingly, as in the case of the LjNPF2.9 the disruption of AtNPF2.9 gene leads to a a reduced root/shoot nitrate content ratio and an increased shoot biomass that is not observed in the presence of ammonium and glutamine as N sources [14]. Our analyses of expression of *LjNPF2.9* indicates a preferential expression in *L. japonicus* roots (Fig. 1), where it is confined to the pericycle cell layer and phloematic structures of the root vascular stele (Fig. 2). This pattern of expression is consistent with the conclusion that LjNPF2.9 acts as an hortologue of AtNPF2.9 on the control of the xylem-to-phloem nitrate flux. Although it is accepted that xylem provides the main route for long distance root-to-shoot nitrate transport, several studies have reported significant concentrations of nitrate into the phloem sap (0.59 to 8.1 mM) [60, 61] and a coordinated xylem- and phloem-mediated transport of nitrate has been proposed for a proper distribution of this nutrient at different developmental stages to ensure an efficient plant growth [2].

In legume plants, the nutritional status and consequent nitrogen demand is a signal that might control nodulation through a systemic signalling pathway [23, 24]. In particular, a negative feedback controlled by the general nutritional status reduces the nodulation competence in plants grown under high N conditions [23]. In our experimental conditions the response to progressively increased KNO_3_ external concentrations is not changed in the *ljnpf2.9* mutants when compared to wild type plants (Fig. 9a), indicating that the higher amount of nitrate accumulated in the shoot of the mutant is not altering the dosage dependent inhibitory effect of the external nitrate. This result must be further investigated but could be due to an increased storage of nitrate, a typical plant response to cope with fluctuation of environmental conditions affecting external nutrient concentrations and energy sources [48, 62, 63].

## Conclusions

Present data indicate that the *LjNPF2.9* gene is involved in the downward transport of nitrate to roots and that disruption of this gene function determines a significant increase of the shoot biomass associated to a higher amount of accumulated nitrate. Importantly, this shoot phenotypic trait doesn’t interfere with symbiotic performances in terms of nodule formation and nitrogenase capacities. This is an important result for prospecting studies aimed to the improvement of NUE in legume crops.

## Methods

### Plant material and growth conditions

All experiments are carried out with *Lotus japonicus* ecotype B-129 F12 GIFU [64, 65]. GIFU Seeds were originally provided by P. Gresshoff (University of Queensland, AU) and then propagated in our plant room facilities. Plants are cultivated in a growth chamber with a light intensity of 200 μmol.m^− 2^.sec^− 1^ at 23 °C with a 16 h/8 h day/night cycle. Phenotypic characterizations have been performed as described in Rogato et al. [66]. Solid growth media has the same composition as that of Gamborg B5 medium [67] except that (NH_4_)_2_SO_4_ and KNO_3_ are omitted and substituted by the proper N source at the required concentration. KCl is added, when necessary to the medium to replace the same concentrations of potassium source. The media containing vitamins (Duchefa catalogue G0415) are buffered with 2.5 mM 2-(N-morpholino) ethanesulfonic acid (MES; Duchefa, MIS03.0250) and pH adjusted to 5.7 with KOH. After germination, unsynchronized seedlings are discarded.

*M. loti* inoculation is performed as described in Barbulova et al. [68]. The strain R7A is used for the inoculation experiments, grown in liquid TYR-medium supplemented with rifampicin (20 mg/L).

### Determination of acetylene-reduction activity (ARA)

The Acetylene-Reduction-Activity assay has been described in D’Apuzzo et al. and Calderon et al. [69, 70]. Detached roots with comparable number of nodules are placed in 10 ml glass vials. The vials are sealed with parafilm and injected with 1 ml of acetylene (C_2_H_2_:air = 1:9 v/v) by using an autosampler syringe. After 30 min of incubation at 25 °C, collect 1 ml of sample from the sealed vial, inject this amount through the septa of the gas chromatograph (PerkinElmer Clarus 580) and then measure the area of the obtained peak of ethylene. After the analysis, the nodules are detached from the root samples under the microscope to carefully isolate these from the root material and weight them collectively. The acetylene reduction activity of the nodules is calculated as the amount of ethylene produced per time and mass of nodules (μmols × 1/h × 1/g nod) by using the following formula: ethylene area x nodule weight (g)^− 1^ x t(h)^− 1^ × 4,12 / 8,880,000 where 4.12 are the μmols of ethylene in 1 ml of gas mixture kept at 1 atm at 20 °C.

### Determination of nitrate content

Root and shoot samples are first weighed and then frozen in the − 80 °C. Crude extracts are prepared by grinding the frozen samples with a tissue lyser (Qiagen, 85,220) at 29 Hz/ for 1 min 30 s. The powder is immediately resuspend in H_2_O (6 ml H2O/gr of fresh weight), vortex and centrifuge at 13.000 rpm to recover the supernatant. The colorimetric determination of nitrate content in leaves and roots extracts follows the procedure described by Pajuelo et al. [71]. Two hundred microliters of 5% (w/v) salicylic acid in concentrated sulfuric acid is added to aliquots of 50 μL from the crude extracts and left to react for 20 min at room temperature. NaOH (4.75 ml of 2 N) is added to the reaction mixtures and the absorbance read at 405 nm scored after cooling. A calibration curve of known amount of NaNO_3_ (Sigma, 74,246) dissolved in the standard extraction buffer is used for analytical determinations. Controls are set up without salicylic acid.

### Leaf morphometric analysis

Leaf area is measured with ImageJ software [72]. The procedure or cell sizes measurement of both adaxial and abaxial lamina of epidermis cell layers is the following: first, images of three microscopic field of views (1.0mmx0.7 mm), randomly chosen for each leaf lamina, are acquired under incident light. Then, count of cells is done with Image-Pro Premiere software (Media Cybernetics, Inc., www.mediacy.com) after applying a watershed algorithm [73] in order to separate adjacent ones. The equivalent diameter of a circle having the average cell area is chosen as cell size.

### *L. japonicus* transformation procedures

Binary vectors are elettroporated in the *Agrobacterium rhizogenes* 15,834 strain [74]. *A. rhizogenes*-mediated *L. japonicus* transformation have been performed as described in Martirani et al. [75]. Inoculation of composite plants is described in Santi et al., [76].

### Plasmids preparation

The *pLjNPF2.9-gus*A T-DNA construct containing 1038 bp upstream of the ATG codon has been prepared in the following way: PCR amplified fragment have been obtained on genomic DNA with the specific forward oligonucleotide containing a *Sal*I site in combination with the reverse primer containing the *Bam*HI site (Additional file 1: Table S1). The amplicon has been then subcloned as *Sal*I-*Bam*HI fragments into the pBI101.1 vector [77] to obtain the translational fusion plasmid pSS1.

The plasmid for expression in *Xenopus laevis* oocytes have been prepared in the following way: cDNA prepared from nodule RNA has been amplified with the forward primer containing the *Xba*I site in combination with the reverse primer containing the *Hind*III site (Additional file 1: Table S1). The 1920 bp fragment double digested with *Xba*I and *Hind*III, has been subcloned into the pGEMHE plasmid containing the 5′-UTR and 3′-UTR of the *Xenopus laevis* β-*GLOBIN* gene [78], pre-digested *Xba*I-*Hind*III to obtain pGEMHE2.9. The correct coding sequence of *LjNPF2.9* has been verified by sequencing.

### Functional analysis of LjNPF2.9 in *Xenopus laevis* oocytes

pGEMHE2.9 has been linearized with *Nhe*I and capped mRNA transcribed in vitro using the mMessage mMachine T7-ultra Kit (Life Technologies). Oocytes preparation is described in [79]. Oocytes obtained surgically fromanesthetized Xenopus were defolliculated by a 1 h collagenase treatment (1 mg.ml − 1, type IA, Sigma Chemical, Saint-Louis, MO) in a medium containing (in mM): 82.5 NaCl, 2 KCl, 1 MgCl2, 5 HEPESNaOH (pH 7.4). Stage V and VI oocytes were selected and placed in a ND96-modified medium containing in mM: 2 mM KCl, 96 mM NaCl, 1 mM MgCl2, 1.8 mM CaCl2, 5 mM HEPES, 2.5 mM sodium pyruvate, pH 7.5, supplemented with gentamycin sulphate (50 mg.mL-1). Defolliculated oocytes were injected with 20 ng of complementary RNA (cRNA) and stored in the modified ND96 medium described above. Two days after injection, batches of 10 injected oocytes were incubated in 1 mL of ND96 solution at pH 5.5 supplemented with 10 mM ^15^NO_3_ supplied as K^15^NO_3_ for 2 h at 18 °C. Oocytes were then rinsed five times in 15 mL cold modified ND96 solution. Batches of 2 oocytes were then analyzed for total N content and atom % ^15^N abundance by Continuous-Flow Mass Spectrometry, using an Euro-EA Eurovector elementar analyzer coupled with an IsoPrime mass spectrometer (GV Instruments, Crewe, UK). Oocytes injected with *AtNPF6.3* cRNA and water were used as positive and negative controls, respectively. Results are expressed in pmol.oocyte-1.min-1.

### Quantitative real-time RT-PCR

Real time PCR is performed with a DNA Engine Opticon 2 System, MJ Research (MA, USA) using SYBR to monitor dsDNA synthesis. The procedure is described in Ferraioli et al. [80]. The ubiquitin (*UBI*) gene (AW719589) has used as an internal standard. The oligonucleotides used for the qRT-PCR are listed in the Additional file 8: Table S4.

### LORE1 lines analyses

LORE1 lines 30,071,286, 30,086,034 and 30,007,925 are obtained from the *LORE1* collection [38–40]. The plants in the segregating populations have been genotyped and expression of homozygous plants tested with primers listed in the Additional file 2: Table S2. After PCR genotyping, shoot cuts of the homozygous plants are cultured in axenic conditions and root induction is obtained through 7 days exposure to 0.1 mg/l naphthaleneacetic acid (NAA, Duchefa catalogue G0903).

### Histochemical GUS analysis

Histochemical staining of whole plant and sections material are performed as described by Rogato et al. [81, 82].

### Phylogenetic studies

The evolutionary history was inferred using the Maximum Parsimony method. The most parsimonious tree with length = 17,866 is shown. The consistency index is 0.358838 (0.355701), the retention index is 0.621797 (0.621797), and the composite index is 0.223125 (0.221174) for all sites and parsimony-informative sites (in parentheses). The MP tree was obtained using the Subtree-Pruning-Regrafting (SPR) algorithm with search level 0 in which the initial trees were obtained by the random addition of sequences (10 replicates). This analysis involved 139 amino acid sequences. There were a total of 768 positions in the final dataset. Evolutionary analyses were conducted in MEGA X [83, 84].

### Statistical analysis

Statistical analyses are performed using the VassarStats analysis of variance program (http://vassarstats.net/). For the ratio of the uncorrelated variables (y = x_1_/x_2_) shown in Fig. 5, the expression giving the standard deviation (SD) is the following: [SD(y)/y]^2^ = [SD (weight)/weight]^2^ + [SD (length)/length]^2^. Standard errors reported in Fig. 5 are then calculated SE = SD/(n-1)^1/2^.

## Additional files

Additional file 1: **Table S1.** Nomenclature and clade sub-division of the complete list of LjNPF proteins. (XLSX 15 kb)

Additional file 2: **Table S2.** NPF amino acid sequences in FASTA format. (DOCX 45 kb)

Additional file 3: **Figure S1.** Phylogenetic tree obtained with the maximum parsimony method and based on the alignment of the 53 *A. thaliana* and 86 *L. japonicus* amino acid NPF sequences. (PDF 429 kb)

Additional file 4: **Figure S2. a** amino acid sequence of LjNPF2.9. The ExxER/K motif is indicated in bold; **b** TMHMM (transmembrane helices based on a hidden Markov model) prediction of LjNPF2.9. (PDF 60 kb)

Additional file 5: **Figure S3.** Representative images of wild type and *ljnpf2.9–1* leaves of different corresponding trifolia. Black bar = 2.5 mm. (PDF 800 kb)

Additional file 6: **Figure S4. a** Representative images of wild type and *ljnpf2.9–1* leaves. The red squares indicate the area photographed in panel b. **b** representative area photographed for epidermis cell size and counting analyses. The cells are numbered in red. (PDF 8967 kb)

Additional file 7: **Table S3.** Measures for average cell size determination from epidermis layers of wild type and *ljnpf2.9–1* superior and inferior lamina. (DOCX 17 kb)

Additional file 8: **Table S4.** Oligonucleotides used in the present work. (XLSX 9 kb)

## Data Availability

All the data supporting our findings are contained within the manuscript. Constructs and seeds are available upon request from MC.

## Abbreviations

*A. thaliana*: *Arabidopsis thaliana*
ARA: Acetylene Reduction Assay
CLC: Chloride channel
*gus*A: β-*glucuronidaseA*
kDa: Kilodalton
kg: Kilogram
*L. japonicus*: *Lotus japonicus*
LORE1: Lotus Retrotransposon1
*M. loti*: *Mesorhizobium loti*
N: Nitrogen
NPF: Nitrate/peptide transporter
NRT2: Nitrate transporter
NUE: Nitrogen use efficiency
*O. sativa*: *Oryza sativa*
qRT-PCR: Quantitative ReverseTranscription-Polymerase-Chain-Reaction
SLAC: Slowly activating anion channel
SNF: Symbiotic Nitrogen Fixation
