# Supplementary material for: Disruption of the Lotus japonicus transporter LjNPF2.9 increases shoot biomass and nitrate content without affecting symbiotic performances
Source: BMC Plant Biol. 2019 Aug 30;19:380. doi: 10.1186/s12870-019-1978-5 (PMC6717371; doi:10.1186/s12870-019-1978-5)
Supplement: Supplementary file 2 — Table S2. NPF amino acid sequences in FASTA format. (DOCX 45 kb) [file 12870_2019_1978_MOESM2_ESM.docx]

>Lj4g3v3061500.1-LjNPF1.1

MDMSMEQSTATEQVTRKKGGYRTMPFIIAIVIFLWNALSNFIPIFGAFLSDAWLGRFHVI

AWGTVIDLIGLIVLWLTAIIRNARPPDCHGESCAGPTGGQLLFLFSSLALMSFGAGGIRP

CSFAFAADQINNPENPQNERIMKSFFNWYYVSVGLSVVVAVVFIVYIQVKAGWIVGFGIP

VGLMLFSSIMFFLGSFMYVKMKPNKSLLTGFAQVIAAAWKKRHLTLPPKNSDMWYFQSGS

SLVQPTDKARYLNKACIIKNREKDLDIHGMAIDPWCMCTERQVEELKAVRKVLPIWSTGI

IIAMSISQHAFSVVQAGTMDRVVRNFEIPATSFGAFAILTLTIWVAIYDRVIVPLLSKYT

KRGLTVKQRLGIGLVLSILALAVAALVERKRRNEAMREGFMNNPKGVVNMSAMWLVPQYC

LTGLAEAFNAIGQIEFYYSQFPKTMSSIAIALFTLGMGVGNLLASLIVKVVKDGTGRRGK

NSWLSSNINLGHYDYYYAFLSLLCFLNLFYFFLCSWAYGSTQDIKVWDEEVDTKLEPEKE

TETE

>Lj1g3v2064310.1-LjNPF1.7

MLTVNVSQGSFLVLQASSMDRHITSNFQVPAASFATFMILSLILWLILYNKVFIPVASKI

KGRQTPLGTKQKMGVGLFSCCISIASLAVVESVRRKIAIEQGYSEQPQAVVNMSAMWLLP

REILDGLAEAANVIGQNEFFLTELPQSMSSVASTLGGLGMSVGSLVASFVLSAVDSVTGG

RGNESWLSSNINKGHYDYYYGIICALSIVNFVYFLYCSKSYGPCKERGK

>Lj3g3v0257310.1-LjNPF1.6

MEKEEMEAALIHEEMAQQHKNPLVQRSKGGFITMPFIIANESLAKLASVGLIPNMILYLT

KDYRVRVVKATQIMYYWLAATNFTPVVGAVIADSYLGRFLAIGLGSILCFLGMAVLWLTS

MSPTARPPPCLHSTEDCQSATTSQLALLLSCFALISIGGGGISCSLAFGADQLKKKTDPD

NQRVLESFISWYIALQAIAVVFSLTGIIYIQDHFGWKLGFGVPAALMLLSTFLFFIISPR

YIKQKPHSSILTSLAQVTVVAYKNRKLAFPPKDSAGMYHHEKDSTLVAPSDKLRFLNKAC

IIKDREKDIASDGSASSTWSLCTIEQVEGLKAIIKVIPLWSSSIMISVSTSQTSFRLLQA

KTMDRHITPSFEIPAGSFGLFSMVSVFITAGVYDRVILPLASKLRGKSVSISAKK

>Lj3g3v2314180.1-LjNPF1.3

MASQNISQKPQRSKGGLVTMPFIIANEALANVATIGLLPNMILYLMGSYNIHMAKATQIN

LLSSAATNVTPVLCAFIADSYLGRFLVVGLGSIVTLLGMALLWLTAMIPQARPSPCNSAT

KTCKPATTKQMTMLISSLALMSIGKGGLSCSLAFGADQVNRKDNNSNKQRALEIYFSWYY

AFVAISVIIALTGIVYIQDHLGWKLGFGVPTALMLLSTIFFFLASPLYVKNQIQDSLITG

FARVIVVAYKNRKLPLPAGNSAGILYHRKKDTNLVVPTDKLRFLNKACLIKDHEKDIASD

GSPSNPWSLCTIDQVEELKAIIKVIPLWSTGIMMVLNIGGSFGVLQAKSMNRHITPNFEV

PPGSFGVIMVITIFLWVTLYDRVIIPIASKLRGKPVRISAKTKMGIGLFFSFLHLVTAAI

VETMRRKRASTEGYINDTHAVLNMSAMWLVPQLFLGGLAEAFNAIGQNEFYYTEFPKTMS

SVASSLLGLGMGAGNVMSSLVFSIVQNVTKRGGKEGWAADNINKGHYDWYYWVLAALSVV

NIFYYLICSKAYGPTVDQISKVTDENGSNEDNGLKEKESG

>Lj3g3v2314200.1-LjNPF1.4

MPFIIANEALARVATLGLLPNMVLYLMGNYHLHLSQATYILLLSVATTNFMPLPGAFIAD

SYLGRFLAVGLGSIITFLGMTLLWLTAMIPQARPPPCNPATESCKSATTEQMTMLISSLA

LMSIGNGGLQCSIAFGADQVNRKDNSDNQRALEIYFSWYYASSAISVIIAFTGIVYIQDH

LGWKLGFGVPAVLMFLSTFFFFLASPIYIKNETHSSLFTDFLRVIVVAYKNRKLRLPSKK

SAGMYHQKKDSDLVFPTDKLRFLNKACFIKDPEKDIASDGSASNPWSLCTIDQVEELKSI

VKVIPLWSTGILMSLNIGGSFGLLQAKSLNRHITRNFEVPAGSLTVIMIFTIFIWIALYD

RVIIPLASKLRGKPVRISAKRRMGIGLFFSFLHLVTAAIVETTRRKRAISEGYINDTHAV

LNMSAMWLFPQLCLGGIAEAFNVIGQNEFFYTEFPKTMSSIASSLFGLAMAVGYVLSSLV

FRIVENVTSRGGKDGWVSDNINKGRYDKYYWVLASLSAVNILYYLVCSWAYGPTADQESK

VTEENDSNEEELPLIE

>Lj3g3v0257320.1-LjNPF1.2

MDKEVELAYAAVEDESQPQRRKGGLITMPFIIANEALARVATIGLLPNMILYLMGTYRLH

LGTATQILLLSSASSNFTPVVGAFIADSYLGRFLAVGLGSAITLLGMALLWLTAMTPQAR

PPTCNHAAESCKSATSGQMAILLSAFALMSIGNGGLSCSLAFGADQVNRKNNSNNPRVLE

IFFTWYYASTTIAVIIALTGIVYIQDHLGWKLGFGIPAALMLLSTVFFLLASPLYIKNKI

QTSLITGFAQVTVAAFKNRKLSLPPKNSAELYHRRKDSDLVIPTDKLRFLNKACVIKDPK

QDITSDGSASDPWRLCTIDQVEELKAIIRVIPLWSSGIMMSLNIGGSFGLLQAKSMNRHI

TSHFEVPAGSFSVILVAVIFLWIALYDRAFLPLASKIRGKPVRISAKRRMGIGLLFSFLH

LVTSATLETIRRKRAIEAGYINNAHGVLNMSAMWLAPQLCLGGIAEAFNAIGQNEFYYTE

FPRTMSSVASSLLGLGMAAGNIVSSFVFSAVENITSSGGKEGWVSDNINKGRFDKYYWVV

VGISGLNLVYYLICSWAYGPTVDQISKVAEENGSKVSGEIGSKEKELTELQNVGQVDKVS

KISEENGSKEEELSELRINGVQDEH

>Lj0g3v0061149.1-LjNPF1.5

MEYSSVADDKLIENSIISSSSEQPRKGGLRTMPFIIVNECLEKVSSYGILPNMILYLRDD

YHMPIAKATNVLYTWSAMSNILSIFGAFLSDSYLGRFSVIFIGSFSSLLGVTILWLTAMI

PVLRPSCELCNSATATQLLLLFLSFGLISIGAGCIRPCSIAFGADQLTIKENSKNERLLD

SYFNWYYTSIGASTIVALGVITYIQENLGWKFGFGVPAALMLISALSFTLGSPFYVKLKP

SKSLLTGFVQVAVAATKNRKLSLPVGNFELYYQDRDSEPIVPTDSLSCLNKACIIRNPET

DLNPDGSVSNPWSLCTVKQMESLKSLLRVLPMWSTGIFMMMTQSSFSTLQAKTMNRRLFG

NFEIPAGSFTVIMVITLSIIIPLYDRVAVPILAKYTGQPRGFSSKTRIGIGLLFVIVAKA

TSAVVETMRRNVAIEEGFEYQPYAVINMSALWLVPEFVLLGIAEAFTPVGQVEFFYSHFP

KSMSSFAMALFTLELAAADVVGSVVVNVVDKVTSLGGNGSWLSTNINRGHLNYYYVLLCF

LGMVNFLYYLAICWAYGPDGGENLEASAGKEDDQFDYKELPTS

>Lj4g3v1604500.1-LjNPF2.5

MQQVEEVKCLLRVLPIWVSGVLYFVVIVQQHTILVFQALLSDRRIGQTKFMIPGASYYVF

LMISVAIWLPIYDRKIVPFLQRLNRKEGGITLLQRMGIGIFFSILSMLVSAMVEEHRRTL

ALTQPLGVETRKGAISSMSGLWFIPQLALAGLAEAFMSVAQVEFYYKQFPENMRSIAGSL

YYCGHAGSSYLSSLLISVIIQITARSESGNWLPEDLNKGRLDNFYRILAVLEIVNLGYFV

LCARWYRYKGTDSSSIELDKVTKQSERNANGVL

>Lj2g3v1349210.1-LjNPF2.9

MLGARDKRTNLSGFPFSSWSLVCCRSGFNSSSSAPQKDLPNNENLTKNCSSESSNKKKPG

GWKAMPFILGNETFERLAAFGLFANFMVYLTREFHLNQVDASNILNIWSGITNFFPLVGA

FISDAYVGRFRTIAFASFSSLLGMIMITLTAWLPKLHPPPCSPQQLASNQCVKASTTHVG

SLGIGLFFLSIGSAGIRPCSIPFGVDQFDPTTDEGKKGINSFFNWYYTTFTVVLLITQTV

VVYVQDSVSWKIGFAIPTLCMFCSIIFFFVGTRIYVHVKPEGSIFSSIAQVLVAAYRKRN

VNLPSEKQVDGVFYDPPLKESAALSKLPFTNQFRILNKAALIMEGEVNPDGSRVNQWNLV

SIQQVEEIKCLARIFPIWAAGILGFTAMAQQGTFTVSQAMKMDRHIGSKFQIPAGSLGVI

SFITIGLWVPFYDRFFVPALRRITKHEGGITLLQRIGIGMVFSVLSMIVAGLVEKVRRGV

ANSNPNPLGIAPMSVMWLAPQLVLMGLCEAFNAIGLIEFFNRQFPEHMRSIANALFSCSF

ALASYVSSILVSTVHHATRTHSHPDWLTNDINAGRLDYFYYLIAGIGVLNLIYFLYVAQG

YHYKGSVELQGKEDVELGSKGELDYYTGPRFEDSK

>Lj4g3v1604490.1-LjNPF2.7

MTMNPTGERMTKRETSENEEESLLKNNETDDDDGDDIKVNYRGWKVMPFIIGNEIFEKLG

AIGTLSNLLVYFTTVFNLENIEATNIINIFNGSTNFATLLGAFLSDTYFGRYKTLAFSTV

ASFLGLLVIQLTAAVEKLHPPHCEQSTTCQGPTEGQMTFLKTGLGLLMIGAAGIRPCNLA

FGADQFNPNTDSGKRGINSFFTWYFFTFTLAQMLSLTIIVYIQSNVSWAVGLGIPAALMF

LSSIIFFVGDKMYVKVKPSGSPITSIVQVIVVAAKKRRLKLPEYLYPSLFSYVAPKSMNS

KLPYTYQFRFLDKAAILTPQDQLNPNGSATDPWNLCSMQQVEEVKCLLRVLPIWVSGVLY

FVVIVQQHTILVFQALLSDRRIGQTKFMIPGASYYVFLMISVAIWLPIYDRKIVPFLQRL

NRKEGGITLLQRMGIGIFFSILSMLVSAMVEEHRRTLALTQPLGVETRKGAISSMSGLWF

IPQLALAGLAEAFMSVAQVEFYYKQFPENMRSIAGSLYYCGHAGSSYLSSLLISVIIQIT

ARSESGNWLPEDLNKGRLDNFYRILAVLEIVNLGYFVLCARWYRYKGTDSSSIELDKVTK

QSERNANGVL

>Lj4g3v1614590.1-LjNPF2.1

MSLISNLTMYLLTSYNLSGIYVVNVVQIWNGSSNIASLVGAFISDTYLGRFHTLLYGTFA

SLLGILTMTLTAAIHQLRPLSCKERPNCQWPHPWQLGILFAGLGLLSIGAGGIRPCNIAF

GADQFDTNTKKGRAQLESFFNWWYFTFTIALIGALTCVVYIQTNVSWTLGFAIPTICLAF

STLIFLFGRHTYIYKKPQGSIFSDLAKVVSAAFRKRKLNASGRTLYDPAPIDLENAPLVQ

TDRFKFLDKAAIIADPSELNNQGMPRNAWRLCSLQQVERLKCLMGILPVWVTGICTFIVM

DQQNTFGVLQVIQTNRSIGPHFKVPPGWMNLISMLALSIWIYIYECIYIPLAKKITKKAT

RLTMEQRIKIGVFLSILCMIVAAIVEKKRRDSAIRHGTFHSPMSFVLLLPQFALSGLNEV

FAAVAIMEFFTLQMPENMRTVAGAVFFLSLSIANYIGSLIVNVVHRVTSQKGRTPWIGGH

DLNHNRLEYYYYLIAALGALNFIYFNFFAKKYLRNKKVQPVDLDMVLEMVGTETKG

>Lj1g3v3443730.1-LjNPF2.2

MSLIANLIVYMHTQYNIENANSVEVFNIWSGFTNFLPLVGAYVADAYVGKFNMLLFGSIA

SLLGMGFMALGAGIPSLRPPSCPTHSDCVHPTGMQLGVLYLGLGLFSIGSGGLRPCNIAF

GADQFDTKTEKGRAQLESFCNWWYFLFTVALLLALTGVVYIQTNVSWFIGFVIPTACFAL

SLTIFMLGQSTYVRMKPKGSIISNFVKVVVAANKKRHVDLKKHSELSFYDPPQPASSESE

PKHTKLAQTNRFRNLDKAAVITDPSERDVNGEPIDGWRLCSVQQVEELKSILTTIPVWVA

GIICFLSMGQGHSFGILQALQTSKSIGPHFIIPPAWMGLVPMIALSMWIFLYEKIYIPWT

MKTTKEGKRLSIEHRILIGIMFSIVSMVVSGLVEVRRRDFALRSGSFESPIGIWWLVPQF

ALSGLVEAFAAIPMMELLTSYWPESVKTLGGAVFFLSISIASYLGTILIRVILVVTNKYG

KTPWLGGNDLNKNRLEYFYYTIAVLGGLNLLYFQFFARSYLRTELVQRPGQNEPEDEENV

HKK

>Lj4g3v1614600.1-LjNPF2.3

MTLTAAIHQLRPLSCKERPNCQWPHPWQLGILFAGLGLLSIGAGGIRPCNIAFGADQFDT

NTKKGRAQLESFFNWWYFTFTIALIGALTCVVYIQTNVSWTLGFAIPTICLAFSTLIFLF

GRHTYIYKKPQGSIFSDLAKVVSAAFRKRKLNASGRTLYDPAPIDLENAPLVQTDRFKFL

DKAAIIADPSELNNQGMPRNAWRLCSLQQVERLKCLMGILPVWVTGICTFIVMDQQNTFG

VLQVIQTNRSIGPHFKVPPGWMNLISMLALSIWIYIYECIYIPLAKKITKKATRLTMEQR

IKIGVFLSILCMIVAAIVEKKRRDSAIRHGTFHSPMSFVLLLPQFALSGLNEVFAAVAIM

EFFTLQMPENMRTVAGAVFFLSLSIANYIGSLIVNVVHRVTSQKGRTPWIGGHDLNHNRL

EYYYYLIAALGALNFIYFNFFAKKYLRNKKVQPVDLDMVLEMVGTETKG

>Lj0g3v0275099.1-LjNPF2.8

MTMNPTGERMTKRETSENEEESLLKNNETDDDDGDDIKVNYRGWKVMPFIIGNEIFEKLG

AIGTLSNLLVYFTTVFNLENIEATNIINIFNGSTNFATLLGAFLSDTYFGRYKTLAFSTV

ASFLGLLVIQLTAAVEKLHPPHCEQSTTCQGPTEGQMTFLKTGLGLLMIGAAGIRPCNLA

FGADQFNPNTDSGKRGINSFFTWYFFTFTLAQMLSLTIIVYIQSNVSWAVGLGIPAALMF

LSSIIFFVGDKMYVKVKPSGSPITSIVQVIVVAAKKRRLKLPEYLYPSLFSYVAPKSMNS

KLPYTYQFRFLDKAAILTPQDQLNPNGSATDPWNLCSMQQVEEVKCLLRVLPIWVSGVLY

FVVIVQQHTILVFQALLSDRRIGQTKFMIPGASYYVFLMISVAIWLPIYDRKIVPFLQRL

NRKEGGITLLQRMGIGIFFSILSMLVSAMVEEHRRTLALTQPLGVETRKGAISSMSGLWF

IPQLALAGLAEAFMSVAQVEFYYKQFPENMRSIAGSLYYCGHAGSSYLSSLLISVIIQIT

ARSESGNWL

>Lj4g3v1119470.1-LjNPF2.10

MASKENKAKKNPHSSPKKQPGGWKAMPYILANETFQRLAIFGLVANFMVYLTRELHLEQV

YAATIVNAWFGFSNFGPLLGAFISDAYVGRFRTIAFGSVVCLLGMMVVTLTSWMTPPPCT

PEQLTLGQCVRASNSQMAVLFTGLGILSIGSAGVAPCSIPFGVDQFDPTTDEGKKGINSF

FNWYYATFTLVLILTQTAVIYIQDSVSWKLGFGIPTLCMFLSIIFFFVGTRVYVYVKAEG

SVFSGVAQVFVAAYKKRKVKYPSQEEINDGVFFDSPFCGTAVSSKLPMTKQYRVLNKAAI

VMEGELNPDGSRVNKWELVSIQQVEEIKCIARIIPVWAAGILSLTSMSQQGTFTVSQALK

MERHLGPNFQIPAGSLGVISLLTIALWIPLYDRFLVPALRKKTKHEGGISLLLRMGIGMV

FSVLSMVVAGSVEKVRRDSAVSNSNAPISVFWLAPQLILMGFFEAFNIIAQIEFFNRQFP

EHMRSIGNSLVSLSFGVSSYVSSIIINTVHHTTGTHDHQDWLTNDINAGRVDYFYYLIAG

ITTLNLIYFIYVARGYQYKGSVQVDLHDHDDVELGSLKG

>Lj4g3v1604470.1-LjNPF2.6

MGESEAGGISMKTIELERVENNEKYPTDEDPKVNYRGWKVMPFIIGNETFEKLGAIGTLS

NLLVYLTTVFNLKNITAANMINIFNGSTNFATLLGAFFSDTYFGRYNTLGFCSLTSFLGL

LLIQLTAGIKNLHPPQCGKESTTCKQPTAGQMTFLLAGFGLLLVGAAGVRPCNLAFGADQ

FNPKTESGKKGINSFFNWYMFTFTFAQMVSLTLIVYIQSNVSWAVGLGIPAALMFIACVL

FFIGTKMYVKIKPSGSPMTSVVQVIVVAIKKRRLKLPAEHPMLSLFDYVSPKCINSKLPY

TYQFRGLDKAAIMTPQDKINPDGSPTDPWNLCSVQQVEEVKCLVRVLPIWFSAILYHLVI

VQQHTILVFQALQSDRRIGHSKFMIPGASYYVFLMLSMTLWLPIYDRIVVPFLSKLTGIE

GGITLLQRMGIGIFLGLLSMIVSGGVEKHRRHLALTDPIGLQPRKGAISSMSGFWLIPQL

TLAGLAETFTAVGQIEFYYKQFPENMKTIGGSLFYCGMAGSSYLSTFLISVVHRTTEKSA

TGNWLPEDLNRGRLDLFYYMIAAIEVMNLGYFLLCSNWYRYKVIDTNKSIVNA

>Lj4g3v1614550.2-LjNPF2.4

MEKNEQKSVEDNEKGVVNNDSKINYRGWKVMPFIIGNETFEKLGTIGTLANLLVYLTTVF

NMSSITATNIINVFNGSANLATLPGAFLTDTYFGRYKTLGFCTFASFLGLLIIQLTALIK

SMHPPHCENESTTCIGPSTGQMAFLLIGFGFLIVGAAGIRPCNLAFGADQFNPNTESGKK

GINSFFNWYFFTFTFAQMVSLSLIVYIQADVSWALGLGIPAALMLFSCIVYFLGSRYYVK

IKATGSPVTSMVQVVVVAVKKRSLNLPEFPLDSSLFTYMSSQSINSKLPHTSQFRFLDKA

AIITSKDQINPDGSASDPWKLCSMQQVEEMKCLLRVIPIWISGMLYYIAIIQQSTMLVFQ

ALQSDRRVFNTNFKIPAASYTIFTMLSLSIWLPIYDRIIVPSLRKLTGKEGGITLLQRMG

IGMFLSVLCMLVSSVVENQRRTMALTKPVVGWEPRKGAISSMSGLWLVPQLALAGLSDAF

TLVGQVEFFYKQFPENMRSLAGSLFFCGLAGSSYLSSLLISIIHRVTEKSASGNWLPQDL

NKGRLDYFYYIITGLQVVNFGYFILCAKWYKYKGVGSAS

>Lj2g3v1155500.1-LjNPF3.1)ì

MENNNDHARSRRKQGGLVTMPFIFANEICEKLAVVGFSTNMISYLTTQLHMPLTKAANTL

TNFGGTASLTPLLGAFISDSYAGKFWTITMASVLYQIGMVSLTISAVLPQLRPPPCRGEE

VCKQATDGQLAVLYISLFLGALGSGGIRPCVVAFGADQFDESDPKQTTKTWSYFNWYYFV

MGAAILVAVTVLVYIQDNVGWGLGLGIPTVAMFLSIIAFIVGYPLYRNVNPAGSPFTRLM

QVAVAAFHKRNVPNVPDPNLLYQNDEMDASISLGGKLVHSEQMKFLDKAAIVTAEDNSKT

PNKWRLNTVHRVEELKSIIRMGPIWASGILLITAYAQQGTFSLQQAKTMDRHITKSFQIP

AGSMSVFTIITMLTTTALYDRVLIRVARRFTGLDRGISFLHRMGIGFVISTIATFVAGFV

EMKRKNVALAHGLIEHSHETIPISVFWLVPQYSLHGLAEAFMSIGHLEFFYDQAPESMTS

TAMAFFWTSISLGNYISTFLVSLVHKFTAGPDGSNWLPDTNLNKGRLEYFYWLITLLQFI

NLIYYLFCAKFYTYKQIQIHDRGDSSSEGNHIELATSETV

>Lj1g3v4082070.1-LjNPF3.4

MEIEKKIVSSVRMTEKEVETPENDNRKQHRRGGIRTLPFILANEACDRFASAGFHSNLIS

YLTQELNMPLVAASNILTNFGGTSSFTPLIGALIADSFAGRFWTITIGSLIYELGLVSIT

VSVLLPNMRPPPCPTQVNCQEATSSQLSILYICLLLTSLGSGGIRPCVVPFSADQFNMTK

DGVASRKWSLFNWYFFIMGFASLSALTIVVYVQDNLGWAWGLGIPTIAMLISIIAFVLGA

PLYKSVKPEGSPLVRLAQVIVAAVKKRNEALPNDCKLLYQNRELDAPIALEGRLLHSDQY

KWLDKAAIVTEEEAKDSKAPPNLWKLTTVHRVEELKSIIRMLPIWASGILLITSSSHIGS

FVIQQARTMDRHLSHSSSFQISPANMSIFSVLTMMIGVMLYERFFVPFVRRFTGNPSGIT

CLQRMGVGFVVNIVATIVSALVEIKRKEVAAKYHLLDDPKAIIPISVFWLVPQYCLHGVA

EVFMSVGHLEFLFDQSPESMRSSATALYCITIAIGSYLGTFLVSLVHNYTGKERNWLPDR

NLNRGRLECYYFLVSGIQVLNFIYYVICAWFYTYKPLEELSERIEEEDLEQADENVSFVN

LKHGKEEEKKQFTKDE

>Lj1g3v4082090.1-LjNPF3.3

MEREEKSMGSEEEAKGKPWRRGGIRTLPFILANELCDRFAVAGFNGNLISYLTQELNMPL

VAAANTLTIFGGTASFTPLIGAIIAESFAGRFWTITIASLIYELGMISVTLSTILPHLRP

PPCPTQVNCQEATSSQLSIFYISLVLISLGSGGIRPCVVPYLGDQFDMTKNGVASRKWNI

FNWYFFFLGFASLSALTIVVYIQDNTGWGWGFGIPTIVMFISIIAFVLGSPFYKNVKPDG

SPLVRLAQVIVAAIKKRNVVLPDNPNLLYQNRELDATISLEGRLLHTDQYKWLDKAAIVT

EEETRDPNAAPNLWKLATVHRVEELKSIIRILPISATGILLIAGSSHLPSFVIQQARTMD

RHLSHSFQISPANMSVFSVLTMMSGLILYERLFVPLARRFTGNPSGITCLQRMGIGFVIN

ILATAISAPVEIRRKEIAAKYNLLDDVKATIPISVFWLAPQYCLHGLADVFMSVGLFEFL

FDQAPESMRSSATAIYCIIIAIGSYAGTFIVSLVHKYSGKDERNWLPDRNLNRGRLECYY

WLVSGIQVLNLVYYGVCVWFYTYKPLEEVAEVMNKEEDLEEVNTKISCVKLKHDGM

>Lj1g3v4082100.1-LjNPF3.6

MLISIFAFVLGAPLYKNVKPEGNPLVRLAQVIVAAVKKRNEALPQDHSLLYQNKELDDAI

SLEGKLLHSDQLRCLDKAAIVSEEEARDPNAQPNLWNIATVHRVEELKCLIRMLPIWASG

ILLKTATSHQQTFVILQARTMDRHLSNSFQIPPASMAIFNVLTMMAGVVVYERAFVPFAR

RFTRNPTGITCLQRMGIGFVFNIAATMVLALVEIKRKAVAAKYHLLDDPNATIPISVFWL

VPQYCLHGVSEVFFFVGHLEFLYDQSPETMRSSATALYSIATAIGHYAGSVLVTLIHNYS

GKERNWLPNRNLNRGRLEYYYFLVSGIQVVNLIYYVICAWFYTYKALDEISEPNMEVELE

QADEESSFINLENSRKTEKREFTKDD

>Lj2g3v1130600.1-LjNPF3.7

MPFIFANDICEKLAVVGFTTNMISYLTTELHMPLTKAANTITNFSGTSSLTPLLGAFIAD

AFAGKFWTITIASILYQIGLICLTLTTVLPQARPPPCKGEEVCQEASGGMLAVLYISLLL

ASLGSGGIRPCVVAFGADQFDPTKTWSYFNWYYFVTGAATLVAVTVIVYIQDNLGWGLGL

GIPAIAMFLSIAAFIVGYPLYRNLNPDGSPFTRLIQVAVAAFRKRKIPDVSDSKLLYQND

ELDASISLGGMLVHSEQMK

>Lj2g3v1141830.1-LjNPF3.2

MNRKFEKMEQDGNHGGKKKGGLITMPFIFANDICEKLAVVGFTTNMISYLTTELHMPLTK

AANTITNFSGTSSLTPLLGAFIADAFAGKFWTITIASILYQIGLICLTLTTVLPQARPPP

CKGEEVCQEASGGMLAVLYISLLLASLGSGGIRPCVVAFGADQFDPTKTWSYFNWYYFVT

GAATLVAVTVIVYIQDNLGWGLGLGIPAIAMFLSIAAFIVGYPLYRNLNPDGSPFTRLIQ

VAVAAFRKRKIPDVSDSKLLYQNDELDASISLGGMLVHSEQMKFLDKAAIVTIEDNCEIP

NKWRLNTVHRVEELKSIIRMGPIWAAGILLITAYSQQSTFSIQQAKTMDRHVTKSFQIPA

GSMTVFTYLTMLTATVFYDRVFVRVARRFTGLDRGISFLHRMGIGFVISIFATFVAGFIE

MKRKNVALAHGLLEHSHETIPISVFWLVPQFSLHGLAEAFMSIGHLEFFYDQAPESMTST

AMALFWASISLGNYGSTFLVSLVHKFTAGPNGSNWLPDNNLNKGRLEYFYWLITLLQIIN

LIYYLICAKLYTYKQTQVHDKGDSSSEEKLIELATADRV

>Lj2g3v1141840.1-LjNPF3.5

MKIGHRFLDKAAIVTIEDNCEIPNKWRLNTVHRVEELKSIIRMGPIWAAGILLITAYSQQ

STFSIQQAKTMDRHVTKSFQIPAGSMTVFTYLTMLTATVFYDRVFVRVARRFTGLDRGIS

FLHRMGIGFVISIFATFVAGFIEMKRKNVALAHGLLEHSHETIPISVFWLVPQFSLHGLA

EAFMSIGHLEFFYDQAPESMTSTAMALFWASISLGNYGSTFLVSLVHKFTAGPNGSNWLP

DNNLNKGRLEYFYWLITLLQIINLIYYLICAKLYTYKQTQVHDKGDSSSEEKLIELATAD

RV

>Lj4g3v2618640.1-LjNPF4.3

MEEELVDGKVDWKGRKALKHKHGGMKVSTLILATFAFENMASFALAVNLLSYFTKIMHYE

LSDAANMVTNYSGVSYMLSIAVAIVADTLIGRYKTVLISGFIECLGLTLLTVQAHFASLK

PPTCNLFDKNAQCEKLSGKNEAFLLIGLYLLASGSAGLKSSLPAHGADQFDERDPKEAKQ

MSSFFNALLLALCIGGAISLTFFVWIQDHKGWDWGFGMSTIAIALGTIVFAFGLPLYRIH

VAQRTNPIIEIIQVYVAAIRNRNLPLPEDPENLYEIEQDKEAVMEIEFLPHRDIFRFLDK

AAIQRESDMELEKPESTSQWKLCRVTQVENAKIILSMVPVFLCTIIMTLCLAQLQTFSVQ

QGYTMDTKITKDFNIPPASLPIIPVIFLIAIIPFYDRICVPLLRKFTGIPTGVTHLQRIG

VGLILSSISMVIAAIIEVKRKAVARDNNLLDAVPLLQPLPISIFWLSFQYFVFGVADMFT

YVGLLEFFYSQAPKGLKATSTCFLWSSMAVGYFLSSILVQVVNSATKNITASGGWLAGNN

INRNHLNLFYLFLSILSLINFFIYLIVSKMYKYRPQEPLVISDDKSEK

>Lj1g3v4808810.1-LjNPF4.8

SSSANTLTNFMGSTFLLSLVGGFISDTYLNRFTTCLLFGSLEVLALTMLTVQAGSDHLHP

DACGKSSCVKGGIAFMFYTSLCLMALGIGGVRGSMTPFGADQFDEKDQIEAKALASFFNW

LLLSSTLGAITGVTGVVWVSTQRAWHWGFFIITIASSIGFVILALGKPFYRIKIPGDSPT

LRIAQVIVVAFKNRKLSLPESQEELYEISDKDAAVEKIAHTNQMRSLDKAAILQDNLKPQ

PWKVCTVTQVEEVKILTRMLPIVASTIIMNTCLAQLQTFSVQQGNVMNLKLGSFTVPAAS

IPVIPLIFISILVPLYELFFVPFARKITHHPSGITHLQRVGVGLVLSSISMAVAGIIEVK

RKDQGRKDPSKPISLFWLSFQYGIFGIADMFTLVGLLEFFYRESPANMKSLSTSLTWLSM

SLGYFLSTVFVNVINAITKRITPSKQGWLHGFDLNQNNLNLFYWFLAILSTLNFFNYLYW

ASRYKYKSEESNSSTGFNGLVEMHLGAKQDWEANGEGTTHPS

>Lj1g3v5059320.1-LjNPF4.10

MKASLLILAFLGLENMGTFSLAVNSVPYFNGVMHYDLADAANMLTNYMGTSYILSIFVAI

LADTWIGRYKSVIYSGFIEFVGLALLTVQARYSSLKPAICNINDPTDHCETLSGSHEAFL

FIGLYLVALGSAGTKAALPSHGADQFDENDPKEAMQMSTFFNTLLLAICLGGSVSLTFIV

WIQINKGWDWGFGIGTIAILLGIIIFAAGLPLYRIRLPQGSNAFTEIIQVFVAALRNRSL

TLPEDPTELYEIGQGEEADLEIEFLPHRDIFRFLDRAAIQENFDEQSNSNSEAPNPWKLC

RVTQVENAKIILGMVPIFCCTIIMTLCLAQLQTFSIIQGYTMDTSFTKHFHIPPASLPII

PVMFLVVIVPIYDRIFVPLLRKFTGIPTGISHLQRIGVGLILSSISMAVAAIIEVKRKRV

AIENNMLDAFPVIQPLPISTFWLSFQYFIFGIADMFTYVGLLQFFYSE

>Lj2g3v1339020.1-LjNPF4.6

MNGVVSCDDHSAQAGAILAVPGSSLNLSPLIFLFSLNPFSESKANNMELEAGQVTRWEGY

VDWRSRPALRGSHGGMLAASFVLGVEILENLAFLANASNLVLYLKQYMHMSPSKSANNVT

NFMGTAFLLALLGGFLSDAFFTSYHVYLISALIEFLGLIVLTIQARSPSLKPPQCDEGTI

CQEVNGGKAAMLFAGLYLVALGVGGIKGSLPAHGGEQFDESTPTGRKQRSTFFNYFVFCL

SCGALIAVTLVVWVEDNKGWEWGFAISTITIFVSIPLFLAGSTTYRNKIPSGSPLTTISK

VLIAAILNCCCTNKNSSNAVVNMVSSPSDPHSGRKESVEETNKASTSAETPSESLKFLNG

AAANKPVFSSLECTVQQVEDVKIVLKVLPVFACTIMLNCCLAQLSTFSVEQAATMNTKLG

SLKVPPASLPVFPVLFIMILAPIYDHVIIPYARRTTKSEMGISHLQRIGIGLVLSIVAMA

VAAVVEVKRKRVATHSGLVDDATKPLPISFLWIAFQYLFLGSADLFTLAGLLEFFFSEAP

IRMRSLATSLSWASLAIGYYLSSAIVSIVNSVTGKGSHKPWLSGANLNHYHLERFYWLMC

LLSGLNFLHYLYWAARYKYRGRGTANE

>Lj4g3v1614700.1-LjNPF4.9

MEEGQVQVWEGYVDWRNRPAKKGHHGGMLAASFVLAVEVLENLAYLANASNLVLYLTKFM

HFSPSTSSNIVTNFMGTAFLLAILGGFLADAFFTTYSIYLISAAIEFMGLLVLTIQAHMP

SLKPPNCLMGDSSSHLCQKLHQGEAVMLFAGLYLAALGVGGIKGSLPPHGAEQLDETTLD

GRKKRSEFFNYFVFSLSCGALIAVTFVVWIEDNKGWQWGLSVSTASILISIPVFLLGSPT

YRTKIPSGSPITSMFKVVVSAVCNNFKSGNSTNAVINMATSPSHTTEIGGEVEESNTTKE

DQLSQ

>Lj4g3v1614700.2-LjNPF4.11

MKQGLLVLTIQAHMPSLKPPNCLMGDSSSHLCQKLHQGEAVMLFAGLYLAALGVGGIKGS

LPPHGAEQLDETTLDGRKKRSEFFNYFVFSLSCGALIAVTFVVWIEDNKGWQWGLSVSTA

SILISIPVFLLGSPTYRTKIPSGSPITSMFKVVVSAVCNNFKSGNSTNAVINMATSPSHT

TEIGGEVEESNTTKEDQLSQ

>Lj6g3v1966840.1-LjNPF4.1

MEKFEVVDSKVEAEIQLVDENKVDWKGRTALKFKYGGMKASLLILAFLGLENMGTFSLAV

NSVPYFNGVMHYDLADAANMLTNYMGTSYILSIFVAILADTWIGRYKSVIYSGFIEFVGL

ALLTVQARYSSLKPAICNINDPTDHCETLSGSHEAFLFIGLYLVALGSAGTKAALPSHGA

DQFDENDPKEAMQMSTFFNTLLLAICLGGSVSLTFIVWIQINKGWDWGFGIGTIAILLGI

IIFAAGLPLYRIRLPQGSNAFTEIIQVFVAALRNRSLTLPEDPTELYEIGQDKEADVEIE

FLPHRDIFRFLDRAAIQENFDEQSNSNSEAPNPWKLCRVTQVENAKIILGMVPIFCCTII

MTLCLAQLQTFSIIQGYTMDTSFTKHFHIPPASLPIIPVMFLVIIVPIYDRIFVPLLRKF

TGIPTGISHLQRIGVGLILSSISMAVAAIIEVKRKRVAIENNMLDAFPVIQPLPISTFWL

SFQYFIFGIADMFTYVGLLQFFYSESPKGLKSTSTCFLWTSMALGYFLSTIIVKCVNGAT

KHTKSGGWLAGNNINRNHVNLFYFFLSIVSLINFCIYLILSKRYKYRPQALTVPNDKSTK

E

>Lj4g3v0012160.1-LjNPF4.2

MPNKAEVSNTTPSAVETLDWRGRPSHPYKHGGIRPAAFLLGLQAFEIMAIAAVGNNLITY

VFNEMHFSLSQSANVVTNFVGTIFLLALLGGYLSDSFLGSFWTILIFGFVELSGFILLSV

QAHLPQLKPPPCNMLITDDGGEHCTEAKGFKAMIFFLAIYLVALGSGCVKPNMIAHGGDQ

FNQEDPKQLNQLSTYFNAAYFAFSLGELVALTILVWIQTHSGMDVGFGISAAVMAMGLIS

LISGTFYYRNKPPQGSILTPIAQVLVAAFFKRKHIYPSNPQMLHGDQNNVGQVHTDKFRF

LDKACIRVEEAGSNTKKSSWRLCSVGQVEQVKILLSVIPIFSCTIVFNTILAQLQTFSVQ

QGSAMDTHLTKSFHIPPASLQSIPYILLIIVVPLYDTFFVPFARRITGHESGISPLRRIG

FGLFLATFSMVSAALLEKKRRDEALNHNKTLSIFWITPQFLIFGLSEMFTAVGLIEFFYK

QSMKGMQAFLTAITYCSYSFGFYLSSILVSLVNKITSNGSSNGGWLHHNNLNQDRLDLFY

WLMAVLSFLNFLSYLFWSNWYSHGSTKSQAESNTKENNPCGQGSKYFE

>Lj5g3v1533390.1-LjNPF4.4

MGDKEVKEWKRKQKGGFRASMFVFALSALDNMGFVANMVSLVLYFIMVMHFDLASSANTL

TNFMGSTFLLSLVGGFISDTYLNRLTTCLLFGSLEVLALVMLTVQAGLDSLHPEACGKSS

CIKGGIAVMLYTSLGLLALGLGGVRGAMVAFGADQFDEKDPVEAKALATFFNWLLLSSTL

GSVVGVTGVVWVNMQKGWHWGFSIITVASSIGFLILALGKPFYRIKAPGDSPILRIVQVI

VVAFKNRNLPLPESNEQLYEAYKDATVEKIAHTNQMRFLDKATILQENFEPRPWKVCTVT

QVEEVKILTRMLPILASTIIMNTCLAQLQTFSVSQGSVMSKKLGSFEVPSPSIPVIPLFF

LCVLIPIYEFIFVPFARKITNHPSGVTQLQRVGVGLVLSSISMAVAGIIEVKRRDQAIKD

PSKPISLFWLSFQYAIFGVADMFTLIGLLEFFYREAPPTMKSLSTSFTYLSMSLGYFLST

VVVSVINTVTKRITPSKQGWLYGSDLNKNNLNLFYWFLAILSCLNFFNFLYWASCYKYKS

EDNSCSKLNVKAVAETTAIMVVDEKKHDKEDMKDHGSTQDLRAKAKESNQTSEANTEGPS

SSDETDDGKQKERNSREWKDR

>Lj0g3v0283739.1-LjNPF4.5

MGICRDMENNPRRQRRLGGNRAALFVYAMEGLENMAFVANAVSLVTYFFGYMNFSLTKSA

TTLTNYMGTAFLLALIGGFICDTYLSRFKTCVLFACMELLGYGLLTVQAHFHQLRPVPCK

DVATTQMSQCKPATGGQAAILYTGLYLVALGTSGVKAALPALGADQFDDKNPKEASQLSS

FFNWFLLSLTIGAIVGVTFIVWISTNQGWYWSFTMSTIAVLFAIISICMGKSLYRNNKPK

GSPLVRIIQVFVAAFKNRKLPIPENEAQLHEIHEKERDDSYEILKKTDQFRFLDHAAIVR

SSAGATTSISWGTWNLCTVTQVEETKILIRMLPIIFSTIFMNTCLAQLQTFSIQQSTTMN

TKIMGFKVPGPSVPVIPLLFMFFLIPLYDRFFVPLARRITGIPTGIRHLQRIGVGLVLSA

ISMAVAGFVETRRKSVAVQHNMVDSTEPLPMSVFWLGFQYAIFGAADMFTLIGLLEFFYA

ESSAGMKSLSTAISWCSVAFGYFTSTVVVVVVNRVSGGWLASNNLNRDKLNYFYWLLSVI

SVVNFGFYLVCASWYKYKTVEDKQGDSKDNVDIAKV

>Lj0g3v0046859.1-LjNPF4.7

MADHDAKEEQRPLNQWRRSKGGFMASMFIFVLSALDNMGFVANMVSIVLYFYGVMHFDLA

SSANTLTNFMASTYLLSLVGGFISDTYLNRFTTCLLFGSLEVLALAMLTVQAASKHLHPE

ACGKSSCVKGGIAVMFYTSLCLLALGMGGVRGSMTAFGADQFDEKDPTEAKALASFFNWL

LLSSTVGAITGVTGVVWVSTQKAWHWGFFIITIASSVGFVTLALGKPFYRIKTPGDSPTL

RIAQVIVVAFKNRKLSLPESHGELYEISDKEATAEKIAHTNQMRFLDKAAIIQENSKPKA

WKVCTVTQVEEVKILTRVLPIVASTIILNTCMAQLQTFSVQQGNVMDLKLGSLTVPAPSI

PVIPLVFISVLVPLYELFFVPFARKITHHPSGITQLQRVGVGLVLSAISMAVAGIVEVKR

RDQGRKDPSKPISLFWLSFQYGIFGIADMFTLVGLLEFFYRESPASMKSLSTSLTWLSTS

LGYFLSTVFVNVINAVSKRITPSKQGWLHGFDLNQNNLNLFYWFLATLSCLNFFNYLYWA

SRYQYKREDSGPGFKPLGEMSLKRVERKQDWEVSQE

>Lj0g3v0363669.1-LjNPF5.1

MEEKAECTQDGTVDFHGQPAISSKTGKWKACAFLVGYEAFERMAFYGVASNLVNYLTTQL

HEDTVSSVRNVNNWSGSVWVTPILGAYIADSYLGRFWTFTLSSIVYVMGMILLTVAVSLK

SLKPTCTNGICNKASTSQIVFFYTALYTTAIGAGGTKPNISTFGADQFDDFNPHEKQTKA

SFFNWWMFTSFLGALIATLGLVYIQENLGWGLGYGIPTSGLLLSLVIFYVGTPMYRHKVR

KTRSPARDLIRVPVAAFKNRKLQLPSDPSQLYEQDMQHYIGSGKRQVYHTPALRFLDKAA

ILEDRTGSTRVPLTVTQVEGAKLIFGMILIWLVTLVPSTIWAQINTLFVKQGTTMDRNLG

PNFRIPAASLGSFVTLSMLLSVPMYDRFFVPFMRRKTGHPRGITLLQRLGIGFSIQIIAI

AIAYAVEVKRMHVIRANHVLGPKDIVPLSIFWLLPQYVLIGIADVFNAIGLLEFFYDQSP

EDMQSLGTTFFTSGIGFGNFLNSFLVTMVDKITGKGESKSWIGDNLNDCHLDYYYGFLLV

LSSLNLLAFLWASSRYIYKRESVRVKEALCVQMEGNPTLDASLGLQV

>Lj1g3v4515810.2-LjNPF5.5

MIVHTRAKAVVFLSSIMSVVDEKGLPSGKEDYTQDGTVDLKGRPVLRSKTGSWKACTFIV

GYELFERMAYYGISSNLVVFLGSKLHEGTVASSNNVSNWAGVVWTTPLVGAYIADAYLGR

YWTFIIASCIYLLGMCLLTLTVSLPALRPPPCAQGVENQDCPQASPWTRGIFYLALYIIA

LGAGGTKPNISTMGADQFDEYEPKENTYKLSFFNWWVFSILVGVLFSTTVLVYIQDNLSW

SLGYGLPTVGLAFSILVFLVGTPFYRHKLPSGSPLTRMLQVFVAAGIKWKAHVPHDPKQL

HELSMEEYANNSRNRIDHTSTLRFLDKAAVKTGKTSPWRLCTVTQVEETKQMTKMVPILI

TTLIPCTMLIQAHTLFIKQGTTLDRSMGPNFEIPPAGLSAILIISMLTSIPIYDRVFVPV

IRRYTKNPRGITMLQRLGAGLLMYVLIMVIAWLTERKRLRVAREKHLLGQHDIIPLSIFI

LIPQYALTGVAENFAEIAKMDFFYYQAPEGMKSLGISYSTTSVALGCFLSSFLLSTVADI

TKKHSHQGWILDNLNISRLDYYYAFMVILSFLNFLCFLVTAKFFDYNVDVTHEKSGSELN

PASLDNARICQGSESTAQPDAK

>Lj1g3v4515810.1-LjNPF5.7

MILHTRQEPIKVDAILSSIMSLVDEEKGLGSRKEDYTQDGTVDLKGGPVLRSKTGSWKAC

FFIVGYELFERMAYYGISTNLVVYLGKKLHQGTVTSSNNVSNWAGVVWTMPLAGAYIADA

HLGRYWTFVIASCIYLWGMCLLTLAVSLPALRPPACAQGVENQDCPQASPLAKGVFFLAL

YIIAVGTGGTKPNISTMGADQFDEFEPKERTHKFSFFNWWVFSILIGVLFAATFLVYIQD

NVGWGVGYGLPTIGLAFSVLLFLVGTPFYRHKLPSGSPITTMLQVFVAAGIKWKAVVPDN

PKQLHELSMEEYVTSRRNRIDHTSSFRFLDRAAIKTGKTSPWMLCTVTQVEETKQMTKMI

PILITTIIPCSLAIQTSTLFITQGTTLDRRMGPHFEIPPAGLSAVMTVFTLISIPIYDCA

FVPAMRQYTKNPRGIAMLQRLGAGLVMYIITMVTAWLSERKRLSVAREYNLLGQHDKIPL

TIFILLPQFALTGVANNFVDIAMLDFFYDQAPEGMKSLGISYSTTSTALGCFFSSFLLST

VADLTKKHGHKGWVLDNLNVSHLDYYYAFMAILSVANFLCFLVAAKLFAYNVDVIHKKSG

LEMNHASLDSARICQSSESTAQPDAKY

>Lj2g3v1022220.1-LjNPF5.15

MPFNFGVKKLTFTCLWCNLTAIAGIERFAFKGVASNLVTYLTDVVGLSNSSAAKMVNSWV

GFTSIMPLLVAPIADAYWHQYSTIMTSSFLYVMGLSALTATALARSWPHRNRTMSSSFLS

LSLYLVSLGQGGYNPSLQAFGADQLGDEEELPSNKNDKSSNKKTLFFQWWYFGVCSGSLM

GVTVMSYIQDTFGWVLGFAIPAISMVISIFIFTCGSPIYLYKEHEDDIQEKKPFMNMFHA

IKASALKCFHCEITLPNDKSETVELELQERPLCQESLKDLNKNSKTCMHLVEQAKVMVRL

LPIWTMLLMFAVIFQQPATFFTKQGMTMKRNIGDFKIPPATLQSAITMSIILLMPLYDRI

FIPIAQMITRQDKGISVMQRMGIGMVLSIIAMVIAALVEMRRLDIGREMRIAGLQSETVP

LSIFWLLPQYILLGISDIFTVVGMQEFFYGEVPKTMRTMGIALYTSVFGVGSFVSALLIT

LVEVYTSSKGVPSWFSDDMVEARLDNYYWLLAWFSGGSLVLYVLLCKFYRYRSDSDSEN

>Lj1g3v1183390.1-LjNPF5.2

MEGKGYTLDGTVDLAGRPVLSSLTGKQKACTYILVYRVLERFAYYGVGANLVNFMTTQLN

KDVVSSITSFNNWSGLATLTPILGAYIADTYTGRFWTITFSLLIYAIGLVLLVLTTTLKS

LRPACENGICREASNLQVALFYTSLYTIAVGSGAVKPNMSTFGADQFDDFRHEEKEQKVS

FFNWWAFNGACGSLMATLFVVYIQEKNGWGLAYSLSAIGFLLSSIIFFWGSPVYRHKSRQ

ARSPSMNFIRVPLVAFRNRKLQLPCNPSELHEFQLNYYISSGARKIHHTSHFSFLDRAAI

RESNTDLSNPPCTVTQVEGTKLVLGMFQIWLLMLIPTNCWALESTIFVRQGTTMDRTLGP

KFRLPAASLWCFIVLTTLICLPIYDHYFIPFMRRRTGNHRGIKLLQRVGIGMAIQVIAMA

VTYAVETQRMSVIKKHHIVGPEETVPMSIFWLLPQNIILGVSNAFLATGMLEFFYDQSPE

EMKGLGTTLCTSCVAAGSYINTFLVTMIDKLNWIGNNLNDSHLDYYYAFLFVISALNFGV

FLWVSSGYIYKKENTSTTEVHDIEMSAEKTVKY

>Lj1g3v1183500.1-LjNPF5.3

MFKSSSLRFLDKAAITESNTDNSNPPCTVTQVEKAKVVIGMFHIWLLMLIPSNFWAVEVT

IFVKQGTTMDRSLGPHFHFPAASLWSFAVFTMVISLPIYDNFFIPFMRRHTGNHRGVKLL

QRVGIGIAIQIIGIAVTYAVEIQRMHVIRKHNIVGPKEVVPMSIFWLLPQNVIFGVANTF

LASGLLEFFYDQSPEEMKVLGTTFYTSTMAGGNYFNSFLVTVVDKVTRKMCDHSWIGDNL

NESHLDHYYAFLFVIAIFNFGVFLWVSNGYIYKKECTSTTEPNDGFCT

>Lj1g3v3443680.1-LjNPF5.13

MPLLGGFIADAYLGRYATVFASSIVYLIGLVLLTLSWFMPGFKPCDDHTNMCTEPRRVHE

VVFFLAIYLISLGTGGHKPSLESMGADQFDEDHVEERKQKMSFFNWWSCALCTGLILGVT

LIVYIQDSINWGVADIIFTVVMAFSLLILVIGRPFFRYQKPTGRPLTPMLQVLVAAFSKR

NLPCPSDPAELYEHPKSHTTNTRFLCHTEKLKFLDKAATVEHDGNSAEKQSPWKLATVT

>Lj1g3v4515820.1-LjNPF5.11

MLISIVIYDRAFVPLIRRYTNNPRGITMLQRLGIGLVVHIIIMVTACFVERRRLRVAREN

NLLGQHDTIPLTIFILLPQFALAGIADNFVEIAKMEIFYDQAPEGMKSLGTSYFTTSLGL

GSFLSTFLLSTVADITKRNGHKGWVLDNLNISRLDYYYAFMAVLSLLNFLCFLVVAKMFV

YNVDVRHSNPGLEMSTVPAASSHSQDHNAKMISQITPQL

>Lj1g3v4515840.1-LjNPF5.10

MLCTVTQVEETKQMMKMIPILITTCIPSTIIAQTTTLFIRQGTTLDRSMGPHFEIPPACL

TAFINIFMLISVVIYDRVFVPAIRRYTKNPRGITMLQRLGIGLVLHIIIMTTACLVERKR

LGVAREKNLLGQHDTIPLTIFILLPQFGLTGIADTFVDVAKLEFFYDQAPEAMKSLGTSY

FTTTQSIGSFFSTFLLSTVADLTRKHGHKGWILDNLNVSHIDYYYAFLAMLSTINFLCFV

IVAKFFVYNYDVTQTKMDLELEMNTASA

>Lj1g3v4515850.1-LjNPF5.9

MGIFFFALYIIAAGTGGTKPNISTMGADQFDDFEPKERHHKLSFYNWWVFYILIGTISAQ

TALVYIQDNVGFALGYGIPTIGLAISILVFLLGTPLYRHRLPSGSPLTRMVQVIVAAMSK

WKVKVPDDPKELHEVSTEEYASKGRNIIDHSSSMKFLDKAATKTGQTSPWMLCTVTQVEE

TKQMMKMIPILITTCIPSTIIAQTTTLFIRQGTTLDRSMGPHFEIPPACLTAFINIFMLI

SVVIYDRVFVPAIRRYTKNPR

>Lj3g3v1876180.1-LjNPF5.12

MADSSNSKSNPLIQLITPSPTKGGWHAAIFIIFVEFAERFAYQGLAGNLITYLTNVLNEP

ITTAAKNVNTWVGVSSLFPLLGGFVADSYLGRFNTIVMSSLIYLLGMIFLTLSVSALKSK

TLFFVALYVLSIGDGGHKPCVQTFAADQFDEDSPEEKEAKSSFFNWWYLGIVAGSTAAVF

VVIYLQDNVGWGVGLGVLAGMLALALALFLLGIKRYRKEGPAGSPFTRLAQVFVAAARKR

RVQVTPGGHNNYCYTEVERDDEPHRLHLRPKIHTLLHTPQCRFLDKAAIIDEADTTSKTR

DPWRLCSVTQVEEVKLVLRLIPIWLSCLMFTVVQANLHTYFTKQGSTLVRSIGPHFQIPP

ASLQGLVGVTILFAVPLYDRVFVPLARKFTGQPTGITVMQRIGAGLFLSILNMAVSALVE

TKRVGVARDHGLLDNPKAVLPMSIWWLLPQYTITGVSDAFTIVGLQELFYDQMPESLRSL

GAAAYISIVGVGSFASNVVIAVVEAVSSRHGEKWLGNNLNRAHLDGFYWVMAVMSAVNLG

AYLWLAKAFVYKKVDGGGEITTCQGSRLNRISSEM

>Lj3g3v3754380.1-LjNPF5.16

MEPLTTKVNPPSDPNLGHGNLLALSSAWKWLRGWHITGFRRISYRISQVHSTRIPPPLLR

MSISGQVNIWSGPASLLPLFGAFLADSLLGRYRTIILASLIYILGLGLLTLSALLPSLTK

YECQLDSKFTSCSPQLQVILFFISLYLVAIGQGGHKPCVQAFGADQFDERHPKEHKARST

FFNWWYFTMCAGCMATLWILNYIQDNLSWVIGFGIPCVAMIIALLVFLLGTTTYRFNIQE

CDKISPFLRIGRVFLAAIRNRQTTISSMSIEEESRGILPHQNSEQFNFLNKALLAPKGSK

EEETCSLTEVEEAKAVLRLVPIWATTLVYGIIFAQIFTFFTKQGATMERTIFPGFDIPAA

SLQTLGTLAIVIFSPIYDRLFVPMARAITGKHSGITMLQRIGTGIFISIVTIVLAALVEM

KRLKTAQESGLVDDPGATVPMSIWWLIPQYFLFGVSEVFTMVGLQEFFYDQVPNELRSMG

LALYLSIFGVGSFLSGFLISVIETVTGKDGQDSWFANNLNKAHIDYFYWLLAGFSVVGFA

MFMCFAKSYVYNHKGASRV

>Lj4g3v1155580.1-LjNPF5.18

MLQRIGTGIFISIVTIVLAALVEMKRLKTAQESGLVDDPDAIVPMSIWWLIPQYILFGVS

EVFTMVDLQEFFYDQVPNELRSTGLALYISIFGVGSFLSGFLISMIETVTGKDGQDSWFA

NNLNKAHIDYFYLLLAGFSVVGFAMFICFAKSYIYNQKGITR

>Lj5g3v1174520.1-LjNPF5.4

MEDGRVDEEYTEDGTVNLKGKPVLRSKTGGWKACSFVVVYEVFERMAYYGISSNLILYLT

KKLHQGTVASANNVTNWVGTIWITPILGAYVADAYLGRYWTFVIASTIYLSGMSLLTLAV

SLPSLKPPQCLETDVTKCKPASTLQLAVFYGALYTLAVGTGGTKPNISTIGADQFDDFHP

KEKSHKLSFFNWWMFSIFFGTLFANTVLVYIQDNVGWTLGYALPTLGLAVSILIFLAGTP

FYRHKLRAGSTFTRMAMVIVAAFKKWKVDVPRDPKELYELDVDEYAKKGSYRIESTPTLR

FLDKACVKTGSTSPWMLCSVTQVEETKQMLRMVPILVATFVPSTMIAQINTLFVKQGTTL

DRHIGSFKIPPASLGAFVTVSLLVCVVLYDRFFVNIMKKFTKNPRGITLLQRMGIGMVIH

IVVMITASVTESYRLRVAKDHGVVESGGQVPISIFILLPQFILMGTADAFLEVAKIEFFY

DQAPETMKSIGTSYSTTTIALGNFISSFLLSTVSRVTREHGSRGWILNNLNESHLDYYYA

FFAVLNFLNFIFFLVVSRFYVYKAEVSDSIEVLAKELKEKTVSNYVIPRD

>Lj6g3v0026660.1-LjNPF5.6

MCLLTLSVSLPSLKPPQCHEINVTKCEKASTLQLAVFYGALYTLAVGTGGTKPNISTIGA

DQFDDFDPKEKRHKLSFFNWWMFSIFIGTLFANSVLVYVQDNVGWTLGYGLPTLGLAISI

IIFLAGTPFYRHRLPTGSPFTRMAKVIVAAIRKWKVPLPSDPKELYELDLEEYAKQGKVR

IDSTPTLRFLNKACVKTGTCTNAGVLCPVTQVEETKQMLRMIPILIATFIPSTMVAQIST

LFVKQGTTLDRGIGNFNIPPASLGTFVTLSMLVSVVLYDRFFVKIMQRLTKNPRGITLLQ

RMGIGVIIHIVIMVVAALTERYRLSVAKQHGLVESGKQVPLSIFILLPQFILMGTADAFL

EVAKIEFFFDQAPESMKSLGTSYSMTSLDIGNFLSTFVLSTVSHVTKEHSHQGWVLNNLN

ASHLDYYYILLAILNFLNFIFFIIVSKFYVYRAEVSDSIKVLGEELKERSVIVSNQVIPK

D

>Lj6g3v0026670.1-LjNPF5.8

MCLLTLSVSLPSLKPPECYEKDVTRCKEASTLQLAVFYGALYILAVGTGGTKPNISTIGA

DQFDDFDPKEKAQKLSFFNWWFSSITIGTLFAICALVYIQDNVGWTLGYGLPTIGLAIAI

IIFLAGTPFYRHKILSGSPFTKMAKVIVASVRKWKVPLPRDSKELYELDLEEYAKEGTFR

IKPTPTLRFLNKACVITGSSTSSSKWVLCSVTQVEETKQMLRMIPILVATFLPSTIFAQI

NTLFVKQGTTLDRGIGKFNVPPASLIAFACLSFLITVVLYDRFFVKIMQRLTKNPRGITL

LQRMGTGIIIHIVNIIVAALIERYRLSVAKQHGLVENGGQVPLSIFILAPQFTLMGIANA

F

>Lj2g3v1022220.2-LjNPF5.14

MASKRLSKSCILLIAIAGIERFAFKGVASNLVTYLTDVVGLSNSSAAKMVNSWVGFTSIM

PLLVAPIADAYWHQYSTIMTSSFLYVMGLSALTATALARSWPHRNRTMSSSFLSLSLYLV

SLGQGGYNPSLQAFGADQLGDEEELPSNKNDKSSNKKTLFFQWWYFGVCSGSLMGVTVMS

YIQDTFGWVLGFAIPAISMVISIFIFTCGSPIYLYKEHEDDIQEKKPFMNMFHAIKASAL

KCFHCEITLPNDKSETVELELQERPLCQESLKDLNKNSKTCMHLVEQAKVMVRLLPIWTM

LLMFAVIFQQPATFFTKQGMTMKRNIGDFKIPPATLQSAITMSIILLMPLYDRIFIPIAQ

MITRQDKGISVMQRMGIGMVLSIIAMVIAALVEMRRLDIGREMRIAGLQSETVPLSIFWL

LPQYILLGISDIFTVVGMQEFFYGEVPKTMRTMGIALYTSVFGVGSFVSALLITLVEVYT

SSKGVPSWFSDDMVEARLDNYYWLLAWFSGGSLVLYVLLCKFYRYRSDSDSEN

>Lj0g3v0170009.1-LjNPF5.17

MLIALIVFSLGTWNYRFSIRGDQQGPFRRIGRVFIVAANNWRTTPSPTSIEEEAHHATLP

YQGSEQFSFLNKALIASEGSKEEGKVCTVAEVEDAKAVLRLIPIWATSLIFAIVFAQSST

FFTKQGVTMDRKILPGFYVPAATLQSFISLSVVVFIPVYDRIIVPLARVFTGKPSGITML

QRIGTGMFLSIISMAIAAFVEMKRLKMARDHGLIDMQDVTIPMTIWWLIPQYVLFGVADV

FTMVGLQEFFYDQVPDELRSVGLAFYLSIFGVGSFLSSFLISAIQKGTSKDGHDSWFASN

LNRAHLDYFYALLAVLSAVYLTVFWFFSNCYVYKGASR

>Lj1g3v3329140.1-LjNPF6.1

MDTSEIKSPEGPQQGTPGSTSMSRKKLGIYFIESEDRRMAFGRGYTAGSTPVDIHGKSIV

DLSKTGGWIAAFFIFGNEMAERMAYFGLSVNMVAFMFYVMHRPFTSSSNAVNNFLGISQA

SSVLGGFLADAYLGRYWTIAIFTTIYLAGLTGITLCATMSIFVPNQEECSQLTLLLGQCE

PAKPWQMTYLYTVLYITAFGAAGIRPCVSSFGADQFDERSKNYKDHLDRFFNLFYLSVTV

GAIVAFTAVVYVQMKFGWGSAFGSLAIAMGVSNMVFFIGTPLYRHRLPGGSPLTRVAQVL

VAAFRKRKAAFVSSDFVGLYEVPGRRSAIKGSGKIAHTDDFRFLDKAALQLKEDGANPIP

WRLCTVTQVEEVKILLKLIPIPACTIMLNVILTEFLTLSVQQAYTLNTHLGHLKLPVTCM

PVFPGLSIFLILSLYYQIFVPLFRRITGHPHGASQLQRIGIGLGVSILSVAWAAIFERFR

RNYAIEHGFEFNFLSAMPNLSAYWLLIQYCLIGVAEVFCIVGLLEFLYEEAPDAMKSIGS

AYAALAGGLGCFAATIINSIIKSLTGKEGKESWLAQNINTGRFDYFYWILTALSLVNFCI

FIYSAHRYKYRTQQVYEMEKHDVANNVSSTRVDS

>Lj2g3v2899930.1-LjNPF6.7

MVMVASNGEKKEAEDAVNFRGHPADKSKTGGWLAAGLILGTELAERICVMGISMNLVTYL

VGDLHLHSANSATIVTNFMGTLNLLGLLGGFLADAKLGRYLTVVISATIAAVGVFLLTLA

TTLPSMIPPPCSAVRRQHHECIEASGKQLSLLFAALYTIAVGGGGIKSNVSGFGSDQFDI

TDPKEERNMIFFFNRFYFFISIGSLFSVIVLVYVQDNIGRGWGYGISGGTMLVGVAVLLC

GTPIYRFKKPRGSPLTVIWRVLILAWKKRTHPIPSQPTLLNGYLESKVPHTERLRFLDKA

AILDENSSKNGSKENIWMVSTVTQVEEVKMVIKLLPIWSTCILFWTVYSQMNTFTIEQAT

FMNRKVGSAEIPAGSLSAFLIITILLFTSLNEKLIVPLARKLTDNVQGLTSLQRVGIGLV

FSFVAMMVSAIVEKERRENAVKKHTNISAFWLVPQFFLVGAGEAFAYVGQLEFFIREAPE

RMKSMSTGLFLATLSMGYFVSSLLVSIVDKVSQKKWLKSNLNKGRLDYFYWLLAALGILN

FILFIVLARRHQYKVQHNIEPEDSVDKELVMANEVKIGVDGKEEA

>Lj2g3v2014180.1-LjNPF6.2

MPHLPNPSLGTKIFTSPLTSKMSTLPQTQGQTIQDAWDYKGRPAERSKTGGWTSAAMILG

IEGCERLTTLGIAVNLVTYLTGTMHMGNASSANVVTNFMGTSFMLCLFGGFLADTFIGRY

LTSAIFAAIQAAGVTILTISTIIPGLHPPRCKEESSESCEPANTAQLMVLYLALYTIALG

TGGVKSSVSGFGSDQFDETDEGEKKQMVMFFNWFFFFISIGSLAAVTILVYIQDHLGRDW

GYGICATTILVFLAVFLAGTRRYRFKKLVGSPLTQIAVVYVAAWRKRRLELPSDPPLLYN

VHDIDDETLRKKKQMLPHSKQFRFLDKAAIKDPKIDVKTATERKWYLSTLTDVEEVKLVQ

RMLPIWATTIMFWTVYAQMTTFSVSQATTLDRHIGKSFQIPAGSLTLFFVGSILLTVPIY

DRIIVPIAKKLLKNPQGLTPLQRIGVGLVFSILAMMAAALTEVKRLHVARAHGLGDNPDS

VLPMSVFWLVPQFFFVGSGEAFTYIGQLDFFLRECPKGMKTMSTGLFLSTLSLGFFVSSL

LVTLVHKLTGHRNAWLADNLNQGKLDYFYWLLAVLSGLNLVVFLVCAKWYVYKDKRLAQE

GIELEDQDTSSHA

>Lj4g3v1273860.1-LjNPF6.5

MSFSAMGTLPTTQGKTIPDAWDYKGRPAERSKTGGWIAAAMILGGEVMERLTTLGIAVNL

VTYLTGTMHLGNAVSANVVTNFLGTSFMLCLLGGFLADTFLGRYLNIAVFAAVQATGVTI

LTISTIIPSLHPPKCSEDHTAPCVQADSNQLTVLYLALYVTALGTGGLKSSVSGFGSDQF

DDSDDEEKKGMIKFFDWFYFFVSIGSLAAVTALVYIQDNVGRIWGYGICACAIVFALVVF

LLGTKKYRFKKPVGSPLTQIAEVVVAAWRKRHLQVPSDSSLLFDDDDGMLDESGKTKKQR

LPHSEQFRFLDRAAIKDTENAGGITKK

>Lj2g3v2002250.1-LjNPF6.3

MATLPTTTRGKTVPDACDYQGLPAERSKTGGWTAATMILGGEVMERLTTLGITVNLVTYL

TGTMHLGNAASANLVTNFVGTSFMLCLFGGFLADTYIGRYLNIVVFAAVQATGVALLTIS

TTVPSLSPPKCMEGTPCVRATNTQLTVLFLALYVTALGTGGVKSSVPGFGSDQFDDSEKE

EKNDMVKFLNWYYFVVNIGSLAAVTVLVYIQDNQGRPWGYGICTCAILFALVVFMLGTKK

YRYKKPVGSPLTQIAEVFVAAWSKRRLQLPSDSSLLFNEEDILDEAGRIKKQRLPHSKQF

RFLDRAAIKDSESTGGITVMRKWYLTTLTDVEEVKLVIRMLPIWATTIMFWTVQVQMMTL

SVSQATTMDRRIGKSFTFPAGSMTVFLIGTILLTVPFYDRFVAPIARKALNNPQGLTPLQ

HTGVALVLSVLSMVAAAFVEIKRLRFAESHGLVHDAKEIIPMSVFWLVPQFAIVGIGEAL

MYLGQLDFFLRECPDGMKTMSMGLFLSSRAFGFFLSSLLVGFVNKMTGPNKPWIANNLNQ

GRLYDYYWLLAALSVVNLFLYLVCANWYVYKDKKLAEDTLQE

>Lj2g3v2900010.1-LjNPF6.8

MGGSSLGSSSAVSSFIIQQAKLMNRKVGSLDVPSGSLPAFVIITILLLTSLNEKLTVPLA

WKFTHNIHGLTSLQRIGIGLVCATVAMVVAAIAEKERRDNAVKNHTIISAFWLVPQHFLV

GTGQAFAYVGQLEFFIREAPEGMKSMSTGLFLTAISMGYFVSSLLVSIVDKLSKKKWLKS

NLNKGRLDYFYWLLAVLGVLNFILFIVLAMRHHYKVQHNIEPEDNVDKELVIANEVKIGV

DGKEEA

>Lj2g3v2002190.1-LjNPF6.4

MSFSAMGTLPTTQGKTIPDAWDYKGRPAERSKTGGWIAAAMILGGEVMERLTTLGIAVNL

VTYLTGTMHLGNAVSANVVTNFLGTSFMLCLLGGFLADTFLGRYLNIAVFAAVQATGVTI

LTISTIIPSLHPPKCSEDHTAPCVQADSNQLTVLYLALYVTALGTGGLKSSVSGFGSDQF

DDSDDEEKKGMIKFFDWFYFFVSIGSLAAVTALVYIQDNVGRIWGYGICACAIVFALVVF

LLGTKKYRFKKPVGSPLTQIAEVVVAAWRKRHLQVPSDSSLLFDDDDGMLDESGKTKKQR

LPHSEQFRFLDRAAIKDTENAGGITKK

>Lj2g3v2002200.1-LjNPF6.6

MLPIWATTIMFWSIHAQMTTFSVSQATTMNCHIGSFEIPAASMTVFLIGTILLIVPFYDR

FIAPVAKKVLKNPQGFTPLQRVGVGLVLSVISMVAAAVVELKRLRFAESHGFIDTPKAKM

SLSVFWLVPQFFFVGSGEAFMYMGQLDFFLRECPKGMKTMSTGLFLSTLSLGFFFSSLLV

TIVNKLTGPSQPWIADNLNRGRLHDFYWLLAI

>Lj2g3v2904900.1-LjNPF6.9

MCLLTGATTIPSMKSPPCSSVQRQHHECLEAGGKQVALLLVALYTIAVGAGGVKSSVSGF

GSDQFDTTDPREEKKMVFFFNRFYFFVSTGSLFSVLVLVYVQDNIGRGWGFGIPAGIMLV

SLAFLLYGKPLYRYKRPQGSPLTLIWKVLI

>Lj2g3v2904920.1-LjNPF6.10

MVVAAIAEKERRDNAVKNHTTISAFWLVPQLFLVGTGQAFAYVGQLEFFIREAPEGMKSM

STGLFLTAISMGYFVSSLLVSIVDKLSKKKWLKSNLNKGRLDYFYWLLAVLGVLNFILFI

VLAMRHHYKVQHNIEPEDNVDKELVIANEVKIGVDGKEEA

>Lj2g3v1985190.1-LjNPF7.5

MSCSALPVCQEDHFKEESEITLDGSVDLHGRPAIRAKSGRWVAGIIILLNQGLATLAFFG

IGVNLVLFLTRVVGQNNAEAANNVSKWTGTVYIFSLVGAFLSDSYWGRYKTCAVFQVIFV

IGLMSLSLSSYLFLLKPKGCGKESLQCGKHSSLEMGMFYLSIYLIALGNGGYQPNIATFG

ADQFDEEHSKEGRLKVAFFSYFYLALNFGQLFSNTGLVYLEDEGMWALGFWVSAGSAFAA

LVLFLAGTKRYRHFKPSGNPLPRFCQVLVAASRKSRVQMPSNGEDLFNLDTMESSTNANR

KILHTHGFKFLDRAALISSRDVVDQKDGRYNPWRLCPVSQVEEVKCILRLLPIWLCTIIY

SVVFTQMASLFVEQGAVMDSTVFHFRIPAASMSCFDILSVCLFIFFYRRVLDPFVGKLKK

SNSKGLTELQRMGVGLVIAVMAMLSAGIVECYRLRYAKQECTDCKDTSTLSIFWQIPQYA

FIGASEVFMYVGQLEFFNAQTPDGLKSFGSALCMTSISLGNYVSSLIVSIVMKISTEDHV

PGWIPANLNKGHLDRFFFLLAALTSIDLIAYIACAKWYKNIQLDEKRGGENNEPGSFSV

>Lj4g3v1151760.1-LjNPF7.4

MSCLEVSKEGKFKEEAEEVTLDGSVDWHGRPAIRAKSGRWVAGTIILLNQGLATLAFFGV

GVNLVLFLTRVLGQDNADAANNVSKWTGTVYLFSLVGAFLSDSYWGRYKTCAIFQGIFVL

GLVFLSLSSYLSLLRPKGCGSELLHCGKHSSLEMGMFYLSIYLIALGNGGYQPNIATFGA

DQFDEEHSKEGYSKVAFFSYFYLALNLGSLFSNTILGYFEDEGIWALGFWVSAGSAFAAL

VLFLVGTPRYRHFKPCGNPLSRFCQVLVAAWRKLGVQMTSNGEDLYVVDEEESSTNSNNR

KIILHTHGFKFLDRAAYISSRDLDDKKGGFYNPWRLCPITQVEEVKCILRLLPIWLCTII

YSVVFTQMASLFVEQGAAMKTTIYHFRIPPASMSSFDILSVAVFIFFYRRVIDPLVGKLK

KTKSKGLTELQRMGIGLVIAVMAMVSAGIVECYRLKYAKHGTSSLSIFWQVPQYALVGAS

EVFMYVGQLEFFNAQTPEGLKSFGSALCMTSISLGNYVSSIIVSIVMKISTQDHMPGWIP

GNLNRGHLDRFFFLLAALTSLDLIAYIAVAKWFKNIQTECKHDEDDKIGNLIKV

>Lj3g3v2739630.1-LjNPF7.7

MRLLPVWLCTLFSSVVFIQMLSLFVEQGATMDRTFLKFQIPPASMTTFDIISTTAFIMLF

DVLIVPLYEKVMKRPPKPLSELQRIGIGLAITIVALTVAGFVERKRLEHADHDKGRETSS

LSIFWLTPQYVLVGVAEAFVYVAQMNFFTAQAPDGLKSLGMGLSMSVSALGSYVANFILT

VVMKITSSHGRPGWVSPNLNEGHLDKFYFLCAFLTGLDLILYIVCAKRYVGISLEKREET

NKEEVVT

>Lj4g3v1152940.1-LjNPF7.6

MSCSALPVCQEDHFKEESEITLDGSVDLHGRPAIRAKSGRWVAGIIILCLATLAFFGIGV

NLVLFLTRVVGQNNAEAANNVSKWTGTVYIFSLVGAFLSDSYWGRYKTCAVFQVIFVIGL

MSLSLSSYLFLLKPKGCGKESLQCGKHSSLEMGMFYLSIYLIALGNGGYQPNIATFGADQ

FDEEHSKEGRLKVAFFSYFYLALNFGQLFSNTGLVYLEDEGMWALGFWVSAGSAFAALVL

FLAGTKRYRHFKPSGNPLPRFCQVLVAASRKSRVQMPSNGEDLFNLDTMESSTNANRKIL

HTHGFKFLDRAALISSRDVVDQKDGRYNPWRLCPVSQVEEVKCILRLLPIWLCTIIYSVV

FTQMASLFVEQGAVMDSTVFHFRIPAASMSCFDILSVCLFIFFYRRVLDPFVGKLKKSNS

KGLTELQRMGVGLVIAVMAMLSAGIVECYRLRYAKQECTDCKDTSTLSIFWQIPQYAFIG

ASEVFMYVGQLEFFNAQTPDGLKSFGSALCMTSISLGNYVSSLIVSIVMKISTEDHVPGW

IPANLNKGHLDRFFFLLAALTSIDLIAYIACAKWYKNIQLDEKRGGENNEPGSFSV

>Lj5g3v1356090.1-LjNPF7.3

MGTNYFFCLIGAFLSDSYLGRYLTCIIFQVLLIIGLVVLSLSTHFLLLEPQGCGQIGLLC

EPHKPVQVAILYISIYLIALGNGAADPALATMGSDQFDEEEPKEQRSKSIFFSYFYVAIN

LGSLVAETVLAYIETAGNWVLGFWICAGSGVVSFLLLLSGTHRYRHIKPNGNPFSRFAQV

LVSSLRKIKFQIPTNGEGLYEFRERDDASMRRIHHTNGLRKKNELLEKGQKPNPGHISES

QVPSFSSQISMRNAFDQKKSMRNATIDK

>Lj5g3v1356090.2-LjNPF7.9

MGTNYFFCLIGAFLSDSYLGRYLTCIIFQVLLIIGLVVLSLSTHFLLLEPQGCGQIGLLC

EPHKPVQVAILYISIYLIALGNGAADPALATMGSDQFDEEEPKEQRSKSIFFSYFYVAIN

LGSLVAETVLAYIETAGNWVLGFWICAGSGVVSFLLLLSGTHRYRHIKPNGNPFSRFAQV

LVSSLRKIKFQIPTNGEGLYEFRERDDASMRRIHHTNGLRVLVYVWIHDGKPQ

>Lj5g3v1356090.3-LjNPF7.2

MGTNYFFCLIGAFLSDSYLGRYLTCIIFQVLLIIGLVVLSLSTHFLLLEPQGCGQIGLLC

EPHKPVQVAILYISIYLIALGNGAADPALATMGSDQFDEEEPKEQRSKSIFFSYFYVAIN

LGSLVAETVLAYIETAGNWVLGFWICAGSGVVSFLLLLSGTHRYRHIKPNGNPFSRFAQV

LVSSLRKIKFQIPTNGEGLYEFRERDDASMRRIHHTNGLRFLDRAAIVSSQEKKRVVRER

PKTKPGAHIRISSSLIFLPN

>Lj5g3v2300320.1-LjNPF7.1

MGFLGSLASAVIAFLAFLSGTPRYRYVKPCGNPVVRVAQVFTAATRKRGVVPAKEDQLFE

LDGSESAIKGSRKILHSEDFKFMDKAATITDKDENSSNNTWRLCTVTQVEEAKCVLRMLP

VWLCTIIYSVVFTQMASLFVEQGDVMNSNIGKFNLPAASMSAFGICSVLVCTGIYCRILV

PLAGRLSGNPKGLSELQKMGVGLIIGMLAMVASGATEI

>Lj1g3v2975920.1-LjNPF7.8

MKSKGITQLQRMGIGLVLAIMAMVAAGLVEQFRLKHATSEEGSSSLSIFWQVPQYVLIGA

SEVFMYVGQLEFFNGQAPDRLKSFGSALCMTSISLGNYVSSLLVAIVMKISARDDDMPGW

IPGNLNKGHLDRFYFLLAALTAADFVIYVAMARWYKYVKFQGNNDEEDINKENIELKV

>Lj4g3v0166210.1-LjNPF8.9

MGSVENDSSVVEVEEPLLQDEESKRYTGDGSVDFKGRPVLKQNTGTWKACPFILGNECCE

RLAYYGIATNLVTYLTQKLHQGNVAAARNVTTWQGTCYLAPLIGAILADSCWGRYWTIAV

FSTIYFLGMCTLTLSASVPALKPAECFGPVCPPATPAQYAVFFFGLYLIALGTGGIKPCV

SSFGADQFDDTDPQERIKKGSFFNWFYFSINIGAVVSSTFVVWVQENMGWGLGFGIPAIF

MALAIGSFFIGTPLYRFQKPGGSPITRMLQVVVASFRKRNVVVPEDSSLLFETPDKSSAI

EGSRKLEHSDELRCLDRAAVVSDAENKSGNYSNLWRLCTVTQVEELKILIRMFPIWATGI

VFSAVYAQMSTLFVEQGTMMNTHIGSFNIPPASLSSFDVISVIFWVPVYDRFIVPIARKF

TGKGRGFSELQRMGIGLFISVLCMSAAAIVEIVRLQLAREHGLVDKPVPVPLNIFWQAPQ

YFLLGAAEVFTFVGQLEFFYDQSPDAMRSLCSALSLLTTSLGNYLSSFILTLVTYVTTRG

GNPGWIPDNLNKGHLDYFFWLLAGLSFLNMLVYITAAKRYKQKKAS

>Lj4g3v0166290.1-LjNPF8.10

MGSTENGSSLVEEALLHLQDEESKRYTGDGSVDFKGRPVLKQNTGTWKACPFILGNECCE

RLAYYGIATNLVTYLTRILHEGNVSAARNVTTWQGTCYLTALIGAVLADSYWGRYWTIAV

FSTIYFLGMCTLTLSASVPALNPAECLGSVCPPATPAQYAVFFFGLYLIALGTGGIKPCI

SSFGADQFDDTDPREKVKKGSFFNWFYFCINIGAIVSSTFVVWVQENRGWGLGFGIPALF

MALAIGSFFIGTPLYRFQKPGGSPVTRMCQVVVASFLKRNVVVPEDSRLLFEIPDKSSAI

EGSRKLEHSDELRCLDKAAVVSDAERKSGNYSNLWRLCTVTQVEELKILIRMFPVWATGI

VFSAVYAQLSTLFVEQGTMMNRSIGSFNIPPASLSCFSLISVIFWVPVYDRIIVPVARKF

TGKEKGFSELQRMGIGLFVSVLCMSAAAIVENTRLRLARELDIVDKPVAVPISILLLVPQ

YLLFGAAEVFTCVGQIEFFYDQSPDAMRSLCTALPLLTVSLGNYLSSFILTIVTYFTTQG

GNPGWIPDNLNNGHLDYFFWLLAGLSFLNMLVYIAAAKRYKQKKAS

>Lj4g3v0166160.2-LjNPF8.11

MGSSWNELLFLEEPLLQDEGDSQYTGDGSVDIRGRPVLKQNTGNWKACPFILGNECCERL

AFFGIATNLVTYLTTKLHQENVSAARNVSIWQGTCYLTPLIAAVLADGYWGRYWTIAVFS

MIYVLGLCVLTLSASLPALKPAECFGSACPSATPAQYAVLYFALYVIALGTGGVKSCVSC

FGADQFDDTDPNERIGKASFFNWFYFSIDLGAVVSCSFIVWVQDNAGWGLGFGIPAFFMG

IAIGSLFLGTPLYRFQKPWGSPVTRVSQVVLASIRKRNLVVPDDSSLLYEAPNKRLENKG

SRKLMHIDDLRFLDRAAIVSDSESRSGDYTNPWRLCTVTQVEELKILIRVLPIWATGIVF

AAVYAQMSTLFVEQGMMMDTSIGSFNLPPASLATFDVMSVVLWVPVYDRILVPIARKFTS

KESGFSVFQRMGIGHFISVLCMSSAAVVEIARLQLARELDLVDKPVSVPFSVFWQIPQYF

LLGAAEVFTLIGQLEFFYDQSPDSMRTLCSALSLINFSLGNYLSSFILTIVTYFTTQGGN

PGWIPDNLNKGHLDYFFWLLAGLSFLNMLVYIALFFFHLTSSTQALLPLMKPFTQS

>Lj3g3v2681670.1-LjNPF8.6

MAKDAVYAKDGTIDYLGNPAKKRKTGTWTACYFILGHEFCERFTYYGMSTNLVLYFKHQL

HQHSATASKNVADWGGTCYITPLIGALVADAYLGRYLTILYLSVVYVIGMALLTLSASVP

GLKPTCYGKDNCHASHGQSAVCFLSLYLIALAAGGIKPCISSFGADQFDDADEVEKQHKS

SFFNWFFLSINTGGLIAASLMVWIQDNVSWGWGFGIPPLAMAVSGVSFFSGTRLYRNQKP

GGSPITRICQVIVASIRKYDVEVPNDESLLYETKDKVSAIQGSRKLDHSNGLRFFDKSAV

PGNSDNVKDSVNPWRLCTVTQVEELKSVIRLLPIWVTGIIFATVFGQMSNYFVLQGETMD

TNVGNLKFQIPPASVYIFNNLGVIFWVPVYDRIIVPIARKFTGHKNGLTQLQRIGTGLFI

SIFSMLYASTLETVRLGMVKRHKIYELKEVPMSIFWQVPPYFIIGCAEVFTFIGQLEFFY

EEAPDAMRSLCSAFSLLTIGLGQCLSSLLVTIVIKVTTRNGSAGWLPHNLNYGHLDWFFG

LLTVLSVLNFVVFLVVSKFYT

>Lj1g3v0987670.1-LjNPF8.5

MCDANGCHPTSAQTAACFIALHLIALGTGGIKPCVSSFDADQFDEADEKERKSKSSFFNW

FYFSINIGALVASSVLVWIQMNVGWRWGFGVPTVAMVIAIVFFFIGSRLYRLQIPGGSPL

TRICQVIVAACRKFNVQVPEDKSLLHETIDVESVIKGSRKLDHTNNLNLSCWVRDFPA

>Lj1g3v0987680.1-LjNPF8.3

MKDMPNPWGLCTVTQVEELKSIVHFLPVWASLIAFATVYSQMNTMFVLQGNTMDQHIGPH

FKIPSASLSLFDTLSVIFWTPVYDRIIVPFARRFTGHEQGFTQLQRMGIGLVISIVSMVI

AGILEVVRLDMVRRNNYYDLEIIPMSIFWQVPQYFLVGCAEIFTNIGQLELFYDQAPDAM

RSLCVALSLTTNAIGNYISTLLVTIVTKVTTRDGKLGWIPDNLNRGHLDYFYWLLSILSL

LNFHVFLWIAKRYRYKKVA

>Lj4g3v0166160.1-LjNPF8.12

MGSSWNELLFLEEPLLQDEGDSQYTGDGSVDIRGRPVLKQNTGNWKACPFILGNECCERL

AFFGIATNLVTYLTTKLHQENVSAARNVSIWQGTCYLTPLIAAVLADGYWGRYWTIAVFS

MIYVLGLCVLTLSASLPALKPAECFGSACPSATPAQYAVLYFALYVIALGTGGVKSCVSC

FGADQFDDTDPNERIGKASFFNWFYFSIDLGAVVSCSFIVWVQDNAGWGLGFGIPAFFMG

IAIGSLFLGTPLYRFQKPWGSPVTRVSQVVLASIRKRNLVVPDDSSLLYEAPNKRLENKG

SRKLMHIDDLRYLSLHGVLCSSVISFINCLNIWQSIICCMLTFYLILFNMQFV

>Lj4g3v0166170.1-LjNPF8.14

MSSLFVAQGKRMDKNLGSFKIPAASLSTFALIGVIIWVPIYDRGIVPIAKKFTGNVKGFS

DLQRMGIGLFLSIVCMSAAAILESKRLQIAKEFGLVHKNVPVPLSILWQIPQYFLLGAAQ

VFTFVGQHEFFYEQAPTSMRSFCSALALLTNSLGNYLSTLILIIVAFLTTEDGSSGWITD

NLNEGHLDYFFWLLAGLSFLNMLVYIVCARRYKTQKKVYHMSIS

>Lj4g3v0166190.1-LjNPF8.13

MSSMFVAQGKKMDTHLGSFKVPAASLSTFAIIGVIIWVPIYDRGIVPIARKFTGNVKGFS

ELQRMGIGLFISIMCMSAAAIVETKRLQKAKEYGLVHKKVPVPLSISWQIPQYFLLGAAE

VFTFVGQHEFFYEEAPPSMRSVCSALALLTNSLGNYLSTLILTIVGHITRKYGNHGWITN

NLNEGHLDYFFWMLAGLSFLNMLVYIVCAIRYKKQKVYHMSDSP

>Lj6g3v0597030.1-LjNPF8.4

MLPVWATGIIFATVYGQMSTLFVLQGQTMNTHVGNSNFKIPPAALSIFDTLSVIFWVPVY

DWIIVPIARKFSGHKNGLTQLQRMGIGLFISIFAMVAAAILEVIRLRMVRRHNYYELKEV

PMSIFWQVPQYFLIGCAEVFTFIGQLEFFYEQAPDAMRSLSSALSLLTVSLGQYLSSLLV

TIVTKISTKNGGSGWIPDNLNYGHVDYFFWLLAVLSVLNLIVYLPVAKLYTYKRTVGTLR

>Lj6g3v0597040.1-LjNPF8.8

MTEDDNYTKDGTVDYLGNPANKKQTGTWKACPFILGNECCERLAYYGMSTNLVLYFKKQL

NQHSATASKNVSNWSGTCYITPLIGAFLADSYLGRYWTIACFSIIYVIGMTLLTLSASVP

GIKPTCHGEENCHATDGQSAVCFVALYLIALGTGGIKPCVSSYGADQFDDADVVEKEHKS

SFFNWFYFSINIGALIASSLLVWIQDNVGWGWGFGIPAVAMAVAVVSFFSGTRLYRNQKP

GGSPLTRMCQVVVASMRKCGVQAPDDKSLLYEIADTESAIKGSRKLDHTNELSFF

>Lj0g3v0287969.1-LjNPF8.2

MPDIAKKHDTVEDDLYTKDGTININKQPANKKETGDWKACRYILGNECCERLAYYGMKMN

LVNYLQERFHQGNATAARNVTTWAGTCYLTPLLGAFLADSYLGRYWTIASFSTIYVIGMT

LFTFSAILPGLKPSCDGDSCHPTSGQTAACFIALYLIALGTGGIKPCVSSFGADQFDDND

ETERKKKSSFFNWFYFSINIGALVASSVLVWIQTNVGWGWGFGVPAVAMVIAIIFFFIGS

RRYRIQLPGGSPLTRICQVLVAASRNFKVQVPENESLLYETADAESNIIGSRKLEHTTKL

KCLDKAVVATESDSIKDLPNPWRICTVTQVEELKSFICLLPVWASLIAFATVYGQMGTMF

VLQGNTMDQHIGPHFKIPSASLSLFDTLSVIFWAPLYDRIIVPCARKFTGHELGFTQLQR

IGIGLVISIISMTVAGILEVVRLNIVRKNNYYDLETIPLSIFWQVPQYILVGAAEVFTNI

GQMEFFYGEAPDAMRSLCSALSLTTNALGNYVSTLLVIIVTKVTTSSGSLGWIPDNMNRG

HLDYFYWLLTVLSLLNFL

>Lj0g3v0099189.1-LjNPF8.7

MENPSSKLHIKEEMELSTLVSNGRLDLHGRIADKQTTGGWKASPFIIVNEVTERLAFFSI

AVNMVAYLFSEMHQSLPDAATHVTDWIGAAYVLTLLGAFLADAYLGRFRTILIFSAVYAA

GMILLTVSASLDSLRPDKCIVKPCKQASQGQTAFLYGALALIALGTGGIKPCVSSFGADQ

FDEGDEKEVQKKFAFFNWFFFAINMGALLGITVLVYTQEKLGWGWGFGIPTGATFASIIV

LLAGVRYYRYQKPMGSPFTRFLQVIVASIRNHQRGISVGSETPLYEVQTTQSDIIGARKL

PHTPQYRFFDKAAVVITEGETHTHRSNRWRICTVTQVEELKSFIRVLPVWASTIALSVSF

AQLSTFFISQANITDRRLGDSFKIPAGSVPVFSAVNALLLVPLYEKFIVPFLRNRYGHPR

GITSLQRMGVGLFISIFAMASAALVEKKRREHYAQPFSMSVFWLLPQFFLMGSAEVFTYV

GQLEFFYDEATDGTRSISSAMFLAEIGIGSWLSTALVKIVEGASGGQQRGWLRNDLNSSK

LDYFYWILTGVNVINFLVYLAAARCFRGKGALAQVRDEDESMVKFNGVSL

>Lj0g3v0287979.1-LjNPF8.1

MADVIKKHDAEEDDLYTKDGTLDIKKQPANKKETGNWKACRYILGNECCERLAYYGMSTN

LVNYLKERFNQGNAAAAKSVNTWSGTCYLTPLLGAFLADSYLGRYWTIASFSSIYVIGMT

LLTFSAIVPGLKPSCEAGKCHPTSGQTAACYISLYLIALGTGGIKPCVSSFGADQFDDND

ETERKKKSSFFNWFYFSINIGALIASSVLVWIQMNVGWGWGFGVPAVAMVIAIIFFFIGS

RWYRLQLPGGSPLTRICQVIVAASRKLKVQVPENESLLYETTDAESNIIGSRKLEHTNKL

KCLDKAAVATESDSGKGLPNPWRLCTVTQVEELKSFIRLLPVWASLIAFATVYSQMNTMF

VLQGNTMDQRIGPHFKIPSASLSLFDTLSVIFWAPVYDRIIVPYARKFTGNERGFTQLQR

IGIGLVISIISMIVAGILEVVRLDIVRKNNYYDLETIPLSIFWQVPQYFLVGAAEVFPNI

GQMEFFYGEAPDAMRSLCSALSLTTNALGNYVSSLLVTIVTDVTTRNGSLGWIPDNMNRG

HLDYFYWLLTILSLLNFLVYLWIAKRYTYKKVAGNAQ
