# Supplementary figures and images for: Disruption of the Lotus japonicus transporter LjNPF2.9 increases shoot biomass and nitrate content without affecting symbiotic performances
Source: BMC Plant Biol. 2019 Aug 30;19:380. doi: 10.1186/s12870-019-1978-5 (PMC6717371; doi:10.1186/s12870-019-1978-5)

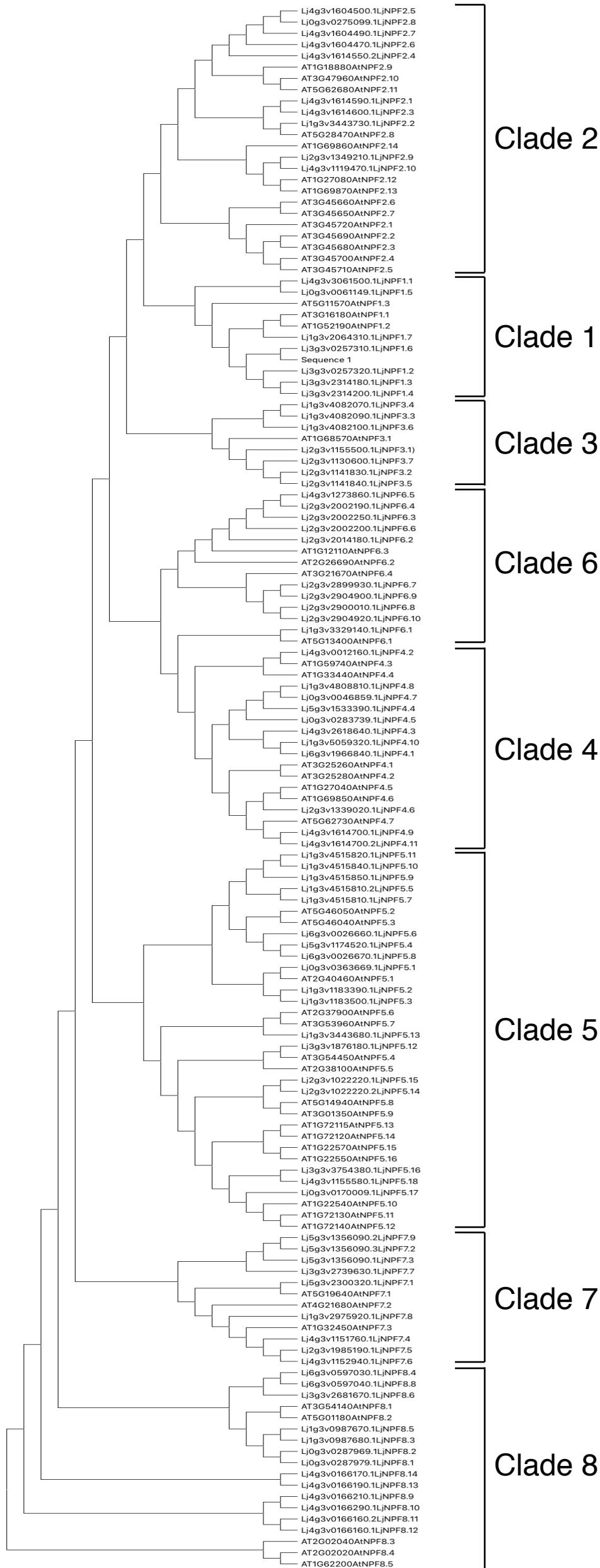

Supplement: Supplementary file 3 — Figure S1. Phylogenetic tree obtained with the maximum parsimony method and based on the alignment of the 53 A. thaliana and 86 L. japonicus amino acid NPF sequences. (PDF 429 kb) [file 12870_2019_1978_MOESM3_ESM.pdf]

**wild type**

***Ljnpf2.9-1***

**1<sup>th</sup> trifolia**

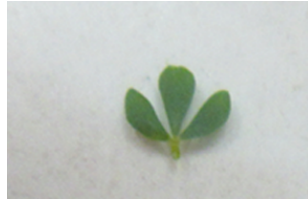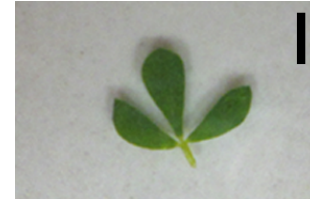

**2<sup>nd</sup> trifolia**

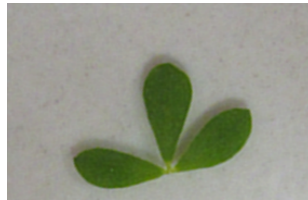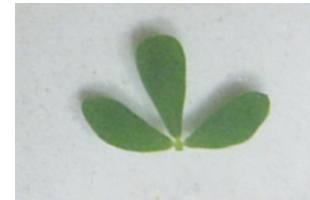

**3<sup>rd</sup> trifolia**

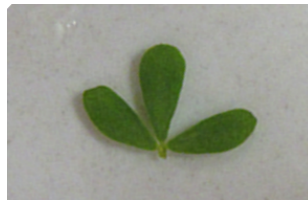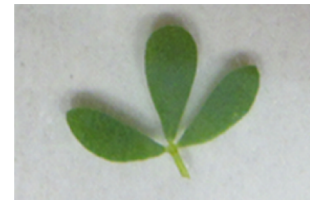

Supplement: Supplementary file 5 — Figure S3. Representative images of wild type and ljnpf2.9–1 leaves of different corresponding trifolia. Black bar = 2.5 mm. (PDF 800 kb) [file 12870_2019_1978_MOESM5_ESM.pdf]

**a** wild type

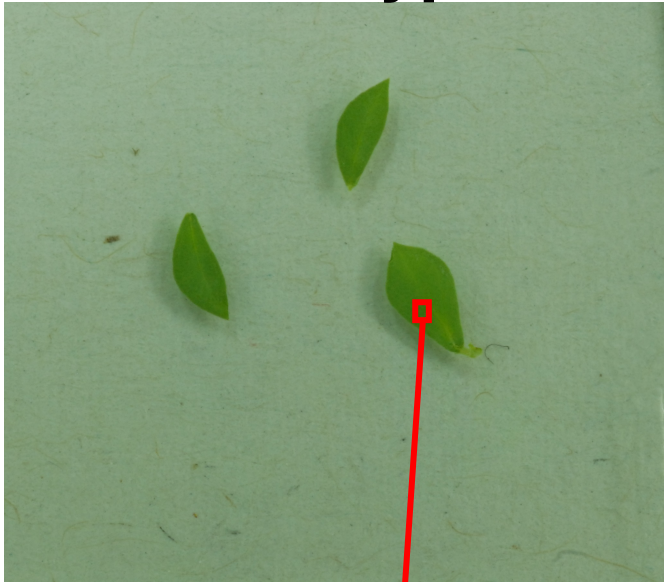

*Ljnpf2.9-1*

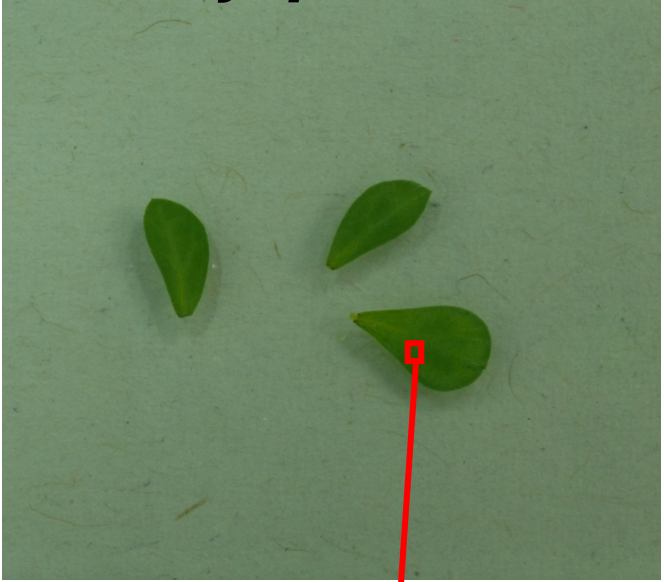

250  $\mu$ m

**b**

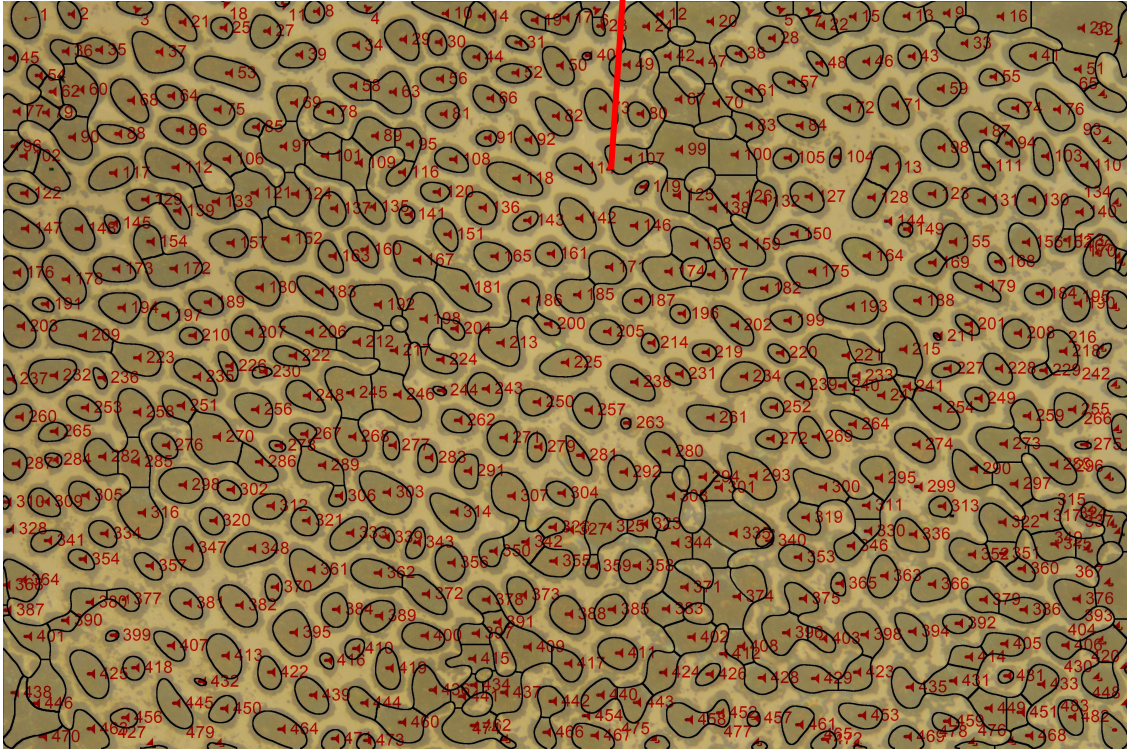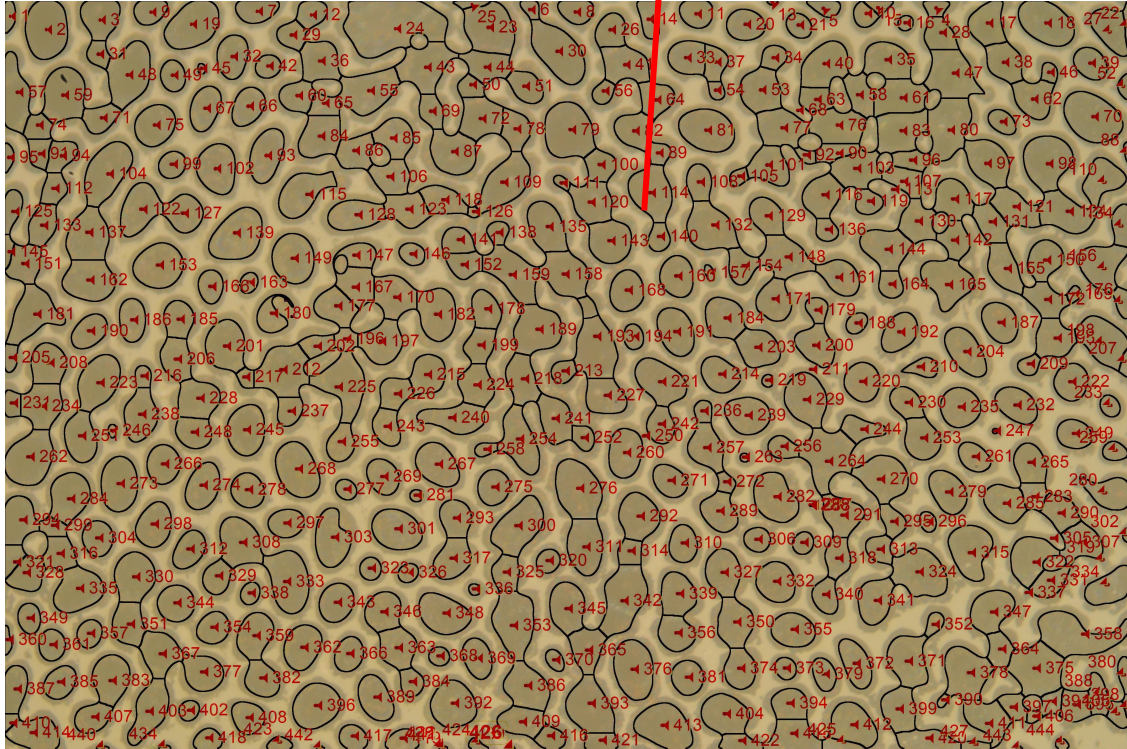

Supplement: Supplementary file 6 — Figure S4. a Representative images of wild type and ljnpf2.9–1 leaves. The red squares indicate the area photographed in panel b. b representative area photographed for epidermis cell size and counting analyses. The cells are numbered in red. (PDF 8967 kb) [file 12870_2019_1978_MOESM6_ESM.pdf]
