# Supplementary material for: Disruption of the Lotus japonicus transporter LjNPF2.9 increases shoot biomass and nitrate content without affecting symbiotic performances
Source: BMC Plant Biol. 2019 Aug 30;19:380. doi: 10.1186/s12870-019-1978-5 (PMC6717371; doi:10.1186/s12870-019-1978-5)

a

MLGARDKRTNLSGFPFSSWSLVCCRSGFNSSSSAPQKDLPNNENLTKNCSSESSNKKKPG
GWKAMPFILGN**E**TF**ERLA**AFGLFANFMVYLTREFHLNQVDASNILNIWSGITNFFPLVGA
FISDAYVGRFRTIAFASFSSLLGMIMITLTAWLPKLHPPPCSPQQLASNQCVKASTTHVG
SLGIGLFFLSIGSAGIRPCSIPFGVDQFDPTTDEGKKGINSFFNWYYTTFTVVLLITQTV
VVYVQDSVSWKIGFAIPTLCMFCSIIFFFVGTRIYVHVKPEGSIFSSIAQVLVAAYRKRN
VNLPSEKQVDGVFYDPPLKESAALSKLPFTNQFRILNKAALIMEGEVNPDGSRVNQWNLV
SIQQVEEIKCLARIFPIWAAGILGFTAMAQQGTFTVSQAMKMDRHIGSKFQIPAGSLGVI
SFITIGLWVPFYDRFFVPALRRITKHEGGITLLQRIGIGMVFSVLSMIVAGLVEKVRRGV
ANSNPNPLGIAPMSVMWLAPQLVLMGLCEAFNAIGLIEFFNRQFPEHMRSIANALFSCSF
ALASYVSSILVSTVHHATRTHSHPDWLTNDINAGRLDYFYYLIAGIGVLNLIYFLYVAQG
YHYKGSVELQGKEDVELGSKGELDYYTGPRFEDSK

b

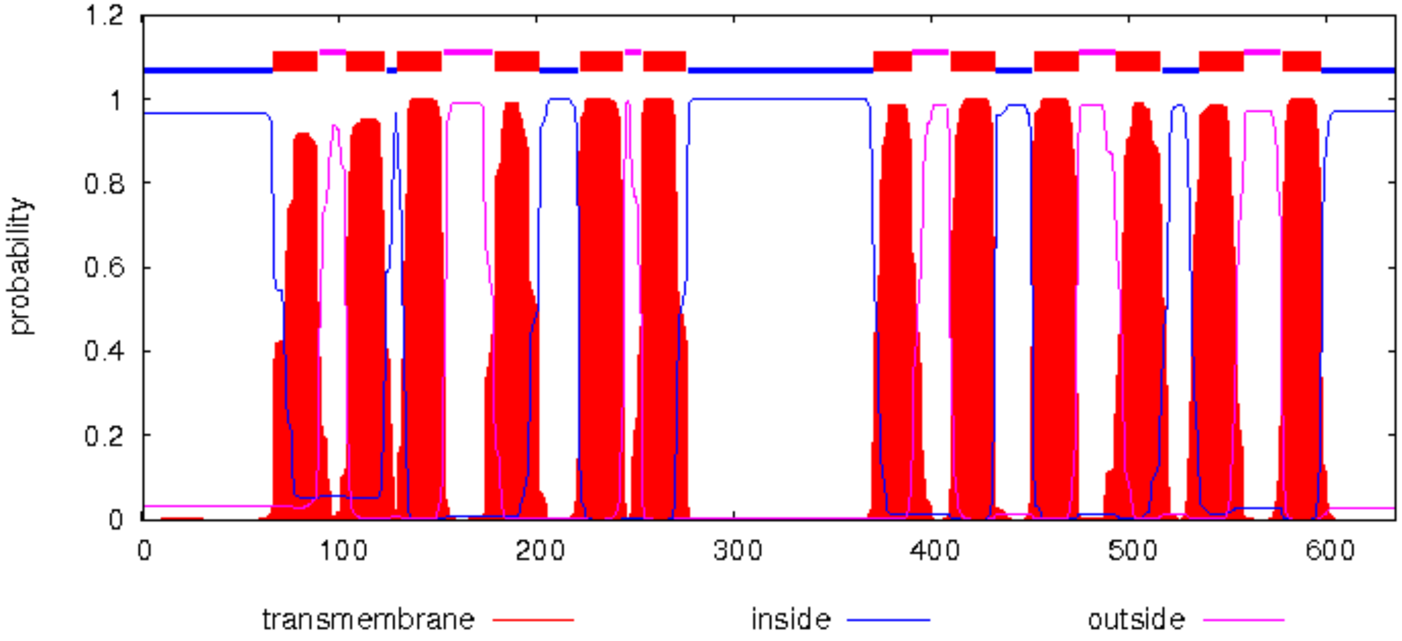

Supplement: Supplementary file 4 — Figure S2. a amino acid sequence of LjNPF2.9. The ExxER/K motif is indicated in bold; b TMHMM (transmembrane helices based on a hidden Markov model) prediction of LjNPF2.9. (PDF 60 kb) [file 12870_2019_1978_MOESM4_ESM.pdf]
