# Supplementary material for: Disruption of the Lotus japonicus transporter LjNPF2.9 increases shoot biomass and nitrate content without affecting symbiotic performances
Source: BMC Plant Biol. 2019 Aug 30;19:380. doi: 10.1186/s12870-019-1978-5 (PMC6717371; doi:10.1186/s12870-019-1978-5)
Supplement: Supplementary file 7 — Table S3. Measures for average cell size determination from epidermis layers of wild type and ljnpf2.9–1 superior and inferior lamina. (DOCX 17 kb) [file 12870_2019_1978_MOESM7_ESM.docx]

**Additional file 7. Table S3**

Average cell size determination, of wild type and *ljnpf2.9-1* leaf epidermis (10 mM KNO_3_).

| FOV 10X |  | Area FOV |  |  |  |  |
| --- | --- | --- | --- | --- | --- | --- |
| 1,02 | mm | 0,69360 | mm^2 |  |  |  |
| 0,68 | mm |  |  |  |  |  |
|  |  |  |  |  |  |  |

SUPERIOR LAMINA

|  |  |  | Measured data by image analysis | |  |  |
| --- | --- | --- | --- | --- | --- | --- |
|  |  |  | WT | MUT7925 | WT | MUT7925 |
| Plant ID | Leaf ID | Image ID | Number of epidermis cells / Area FOV | Number of epidermis cells / Area FOV | Mean diameter (μm) | Mean diameter (μm) |
| 1 | 1 | 1 | 535 | 478 | 40,629 | 42,983 |
|  |  | 2 | 636 | 429 | 37,263 | 45,371 |
|  |  | 3 | 698 | 435 | 35,570 | 45,057 |
|  | 2 | 1 | 539 | 454 | 40,478 | 44,104 |
|  |  | 2 | 575 | 466 | 39,190 | 43,533 |
|  |  | 3 | 526 | 578 | 40,975 | 39,088 |
|  | 3 | 1 | 556 | 533 | 39,854 | 40,705 |
|  |  | 2 | 582 | 463 | 38,954 | 43,674 |
|  |  | 3 | 611 | 370 | 38,018 | 48,855 |
| 2 | 1 | 1 | 487 | 441 | 42,584 | 44,750 |
|  |  | 2 | 585 | 417 | 38,854 | 46,019 |
|  |  | 3 | 499 | 472 | 42,069 | 43,255 |
|  | 2 | 1 | 505 | 482 | 41,818 | 42,804 |
|  |  | 2 | 543 | 541 | 40,328 | 40,403 |
|  |  | 3 | 493 | 574 | 42,324 | 39,224 |
|  | 3 | 1 | 510 | 482 | 41,613 | 42,804 |
|  |  | 2 | 609 | 480 | 38,080 | 42,893 |
|  |  | 3 | 502 | 513 | 41,943 | 41,491 |
| 3 | 1 | 1 | 575 | 456 | 39,190 | 44,008 |
|  |  | 2 | 483 | 394 | 42,760 | 47,344 |
|  |  | 3 | 632 | 468 | 37,381 | 43,440 |
|  | 2 | 1 | 559 | 418 | 39,747 | 45,964 |
|  |  | 2 | 561 | 509 | 39,676 | 41,653 |
|  |  | 3 | 591 | 548 | 38,656 | 40,144 |
|  | 3 | 1 | 570 | 464 | 39,362 | 43,627 |
|  |  | 2 | 563 | 427 | 39,605 | 45,477 |
|  |  | 3 | 528 | 447 | 40,897 | 44,448 |
|  |  |  |  |  |  |  |
|  |  | mean | 557,52 | 471,81 | **39,92** | **43,45** |

INFERIOR LAMINA

|  |  |  | Measured by image analysis | |  |  |
| --- | --- | --- | --- | --- | --- | --- |
|  |  |  | WT | MUT7925 | WT | MUT7925 |
| Plant ID | Leaf ID | Image ID | Number of epidermis cells / Area FOV | Number of epidermis cells / Area FOV FOV | Mean diameter (μm) | Mean diameter (μm) |
| 1 | 1 | 1 | 834 | 337 | 32,541 | 51,191 |
|  |  | 2 | 581 | 347 | 38,987 | 50,448 |
|  |  | 3 | 485 | 315 | 42,672 | 52,949 |
|  | 2 | 1 | 370 | 338 | 48,855 | 51,115 |
|  |  | 2 | 408 | 433 | 46,524 | 45,161 |
|  |  | 3 | 476 | 561 | 43,073 | 39,676 |
|  | 3 | 1 | 451 | 390 | 44,251 | 47,586 |
|  |  | 2 | 401 | 362 | 46,929 | 49,392 |
|  |  | 3 | 410 | 306 | 46,411 | 53,722 |
| 2 | 1 | 1 | 354 | 488 | 49,947 | 42,540 |
|  |  | 2 | 427 | 395 | 45,477 | 47,284 |
|  |  | 3 | 394 | 304 | 47,344 | 53,898 |
|  | 2 | 1 | 366 | 309 | 49,121 | 53,460 |
|  |  | 2 | 613 | 351 | 37,956 | 50,160 |
|  |  | 3 | 435 | 441 | 45,057 | 44,750 |
|  | 3 | 1 | 459 | 346 | 43,863 | 50,521 |
|  |  | 2 | 596 | 354 | 38,493 | 49,947 |
|  |  | 3 | 448 | 336 | 44,399 | 51,267 |
| 3 | 1 | 1 | 387 | 384 | 47,770 | 47,956 |
|  |  | 2 | 458 | 382 | 43,911 | 48,081 |
|  |  | 3 | 523 | 396 | 41,092 | 47,224 |
|  | 2 | 1 | 401 | 414 | 46,929 | 46,186 |
|  |  | 2 | 407 | 421 | 46,581 | 45,800 |
|  |  | 3 | 538 | 353 | 40,515 | 50,018 |
|  | 3 | 1 | 459 | 370 | 43,863 | 48,855 |
|  |  | 2 | 405 | 360 | 46,696 | 49,529 |
|  |  | 3 | 565 | 367 | 39,535 | 49,054 |
|  |  |  |  |  |  |  |
|  |  | mean | 468,56 | 376,30 | **44,03** | **48,81** |

Two factor ANOVA

|  | | |  | |  |  | α = 0,05 | |  |
| --- | --- | --- | --- | --- | --- | --- | --- | --- | --- |
| *Source of variation* | | *SQ* | *dof* | *MQ* | | *F* | *P* | *F crit* | |
| -Plant type | 465,7502 | | 1 | 465,7502 | | 51,26111586 | 1,18997E-10 | 3,932438 | |
| -Lamina | 605,0535 | | 1 | 605,0535 | | 66,59303442 | 8,20747E-13 | 3,932438 | |
| -Interaction | 10,49919 | | 1 | 10,49919 | | 1,155555957 | 0,284876724 | 3,932438 | |
| -In | 944,9271 | | 104 | 9,085837 | |  |  |  | |
|  |  | |  |  | |  |  |  | |
| Totale | 2026,23 | | 107 |  | |  |  |  | |

**Plant types and orientation of lamina are both main source of variation for cell diameter of leaf epidermis without interaction between the factors**.
